# Supplementary material for: Protein tertiary structure modeling driven by deep learning and contact distance prediction in CASP13
Source: Proteins. 2019 Apr 25;87(12):1165–78. doi: 10.1002/prot.25697 (PMC6800999; doi:10.1002/prot.25697)
Supplement: Supplementary file 1 — Supporting Information [file PROT-87-1165-s001.docx]

**Supplementary document**

**Protein tertiary structure modeling driven by deep learning and contact distance prediction in CASP13**

Jie Hou^1^, Tianqi Wu^1^, Renzhi Cao^2^, Jianlin Cheng^1^*

1. Department of Electrical Engineering and Computer Science, University of Missouri, Columbia, Missouri, 65211, USA
2. Department of Computer Science, Pacific Lutheran University, Tacoma, WA 98447, USA

*Corresponding author (chengji@missouri.edu)

**Tables**

| Stage | Network | Optimization | Activation | Nodes in Hidden Layer |
| --- | --- | --- | --- | --- |
| Stage 1 | Network 1 | nadam | Sigmoid | 5 |
|  | Network 2 | nadam | Sigmoid | 15-15 |
|  | Network 3 | nadam | Sigmoid | 5 |
|  | Network 4 | nadam | Sigmoid | 70-25 |
|  | Network 5 | nadam | Sigmoid | 5-5 |
|  | Network 6 | nadam | Sigmoid | 25-15 |
|  | Network 7 | nadam | Sigmoid | 85-15 |
|  | Network 8 | nadam | Sigmoid | 100-70 |
|  | Network 9 | nadam | Sigmoid | 20-5 |
|  | Network 10 | nadam | Sigmoid | 20-15 |
| Stage 2 | Network 1 | nadam | Sigmoid | 5 |

**Table S1.** The configuration of Stage 1 and Stage 2 neural networks of DeepRank.

| Index | Method | Loss | Correlation | Index | Method | Loss | Correlation |
| --- | --- | --- | --- | --- | --- | --- | --- |
| 1 | **MULTICOM_CLUSTER**  **(DeepRank)** | **0.054** | **0.859** | 26 | Wallner | 0.095 | 0.73 |
| 2 | UOSHAN | 0.055 | 0.892 | 27 | ProQ3D-TM | 0.1 | 0.624 |
| 3 | MUFoldQA_M | 0.058 | 0.892 | 28 | VoroMQA-B | 0.1 | 0.601 |
| 4 | Davis-EMAconsensusAL | 0.063 | 0.875 | 29 | MESHI-server | 0.105 | 0.646 |
| 5 | Davis-EMAconsensus | 0.064 | 0.875 | 30 | Bhattacharya-SingQ | 0.106 | 0.564 |
| 6 | ModFOLDclust2 | 0.067 | 0.85 | 31 | MESHI-corr-server | 0.106 | 0.653 |
| 7 | Bhattacharya-ClustQ | 0.068 | 0.819 | 32 | ModFOLD7 | 0.106 | 0.775 |
| 8 | MUfoldQA_T | 0.069 | 0.879 | 33 | MUFold_server | 0.106 | 0.619 |
| 9 | ModFOLD7_rank | 0.071 | 0.756 | 34 | Kiharalab | 0.108 | 0.587 |
| 10 | MULTICOM-CONSTRUCT | 0.072 | 0.814 | 35 | ProQ3D-CAD | 0.108 | 0.639 |
| 11 | RaptorX-DeepQA | 0.073 | 0.818 | 36 | FALCON-QA | 0.112 | 0.632 |
| 12 | SBROD-plus | 0.074 | 0.678 | 37 | MASS1 | 0.112 | 0.6 |
| 13 | ProQ4 | 0.077 | 0.699 | 38 | ModFOLD7_cor | 0.112 | 0.758 |
| 14 | SBROD-server | 0.079 | 0.659 | 39 | VoroMQA-A | 0.112 | 0.624 |
| 15 | SBROD | 0.08 | 0.641 | 40 | Bhattacharya-Server | 0.114 | 0.534 |
| 16 | Pcomb | 0.084 | 0.794 | 41 | MULTICOM-NOVEL | 0.115 | 0.564 |
| 17 | Grudinin | 0.085 | 0.636 | 42 | LamoureuxLab | 0.117 | 0.642 |
| 18 | ProQ3D | 0.087 | 0.639 | 43 | ProQ2 | 0.117 | 0.588 |
| 19 | CPClab | 0.089 | 0.684 | 44 | MASS2 | 0.124 | 0.56 |
| 20 | MESHI | 0.089 | 0.669 | 45 | SASHAN | 0.128 | 0.582 |
| 21 | Pcons | 0.09 | 0.761 | 46 | MUfoldQA_S2 | 0.135 | 0.71 |
| 22 | ProQ3D-lDDT | 0.09 | 0.651 | 47 | PLU-AngularQA | 0.136 | 0.491 |
| 23 | MESHI-enrich-server | 0.091 | 0.671 | 48 | 3DCNN | 0.142 | 0.551 |
| 24 | ProQ3 | 0.092 | 0.576 | 49 | Jagodzinski-Cao-QA | 0.15 | 0.281 |
| 25 | FaeNNz | 0.093 | 0.694 | 50 | PLU-TopQA | 0.212 | 0.045 |

**Table S2.** The results of DeepRank on stage 2 models in the quality assessment category for 72 human targets in comparison with other CASP13 QA methods (note. the results of T0988 and T0994 are not provided in the official CASP13 evaluation: <http://predictioncenter.org/casp13/qa_diff2best.cgi>). DeepRank was registered under the group name “MULITCOM_CLUSTER” in the quality assessment category. It has a lower average loss of model selection than all the other CASP13 quality assessment methods according to the GDT-TS metric.

| Alignment tools | # of times generating best or top 5 models for all domains | | | | | | Averaged GDT-TS for all domains | | |
| --- | --- | --- | --- | --- | --- | --- | --- | --- | --- |
|  | All 112 domains | | 45 FM +  FM/TBM | | 67 TBM +  TBM-hard | | All 112 domains | 45 FM +  FM/TBM | 67 TBM +  TBM-hard |
|  | Top1 | Top5 | Top1 | Top5 | Top1 | Top5 |  |  |  |
| HHsuite ^1 *^ | 53 | 109 | 16 | 44 | 37 | 65 | 55.46 | 33.50 | 70.21 |
| HHblits ^2^ | 13 | 64 | 7 | 27 | 6 | 37 | 49.87 | 30.60 | 62.81 |
| RaptorX ^3^ | 11 | 70 | 8 | 35 | 3 | 35 | 49.63 | 30.18 | 62.90 |
| Alignment Consensus ^4^ | 10 | 62 | 3 | 23 | 7 | 39 | 47.83 | 26.24 | 62.34 |
| MUSTER ^5^ | 9 | 58 | 6 | 30 | 3 | 28 | 47.58 | 28.30 | 60.92 |
| DeepSF | 4 | 23 | 2 | 15 | 2 | 8 | 35.08 | 21.95 | 47.39 |
| JackHMMER^*^ | 3 | 24 | 0 | 7 | 3 | 17 | 43.89 | 23.83 | 54.37 |
| COMPASS ^*^ | 2 | 30 | 1 | 17 | 1 | 13 | 44.68 | 25.22 | 57.75 |
| PSIBLAST ^*^ | 2 | 11 | 1 | 3 | 1 | 8 | 39.33 | 18.65 | 52.25 |
| SAM ^*^ | 2 | 19 | 0 | 2 | 2 | 17 | 42.67 | 20.92 | 57.29 |
| BLAST ^*^ | 1 | 6 | 0 | 0 | 1 | 6 | 39.30 | 16.53 | 51.19 |
| FFAS ^*^ | 1 | 31 | 1 | 12 | 0 | 19 | 45.35 | 23.63 | 59.94 |
| HMMER ^*^ | 1 | 16 | 0 | 1 | 1 | 15 | 46.18 | 21.04 | 56.62 |
| CSI-BLAST ^*^ | 0 | 14 | 0 | 4 | 0 | 10 | 43.89 | 19.89 | 57.55 |
| PRC ^*^ | 0 | 23 | 0 | 5 | 0 | 18 | 44.52 | 22.03 | 57.81 |
| Best of models generated by all methods | | | | | | | 59.36 | 40.75 | 71.86 |
| Best of models generated by alignment algorithms | | | | | | | 57.95 | 37.25 | 71.84 |
| MULTICOM-CONSTRUCT ^6^ | | | | | | | 54.60 | 33.20 | 68.97 |
| MULTICOM-CLUSTER ^7^ | | | | | | | 53.81 | 34.04 | 67.09 |

^1^ Different versions of HHsuite are used in MULTICOM, including HHsearch-1.2, HHsearch-1.5, HHsearch-1.5.1, HHsuite-2.0.8 and HHsuite-3.0. All these tools search a protein sequence against our in-house template sequence/HMM database which is weekly updated from <https://www.rcsb.org/>

^2^ HHblits from HHsuite-3.0 is used to search the protein sequence against the PDB70 template database which are curated by HHsuite and weekly updated from http://wwwuser.gwdg.de/%7Ecompbiol/data/hhsuite/databases/hhsuite_dbs

^3^ The RaptorX program and its template database were updated to latest version that was released before April 30^th^, 2018.

^4^ Our in-house multi-template selection and combination algorithms [1].

^5^ The I-TASSER suite and database were updated to latest version (I-TASSER5.1) that was released before April 30^th^, 2018.

^6^ Our tertiary structure (3D) predictor in CASP13, which uses DeepRank to select best structures.

^7^ Our tertiary structure (3D) predictor in CASP13, which uses APOLLO [2] to select best structures.

* These tools search a protein sequence against our in-house template sequence/profile database which is weekly updated from <https://www.rcsb.org/>

**Table S3**. The statistics of different alignment tools used in the MULTICOM that were able to generate best models or one of best 5 models for 112 domains in CASP13, including 67 template-based (TBM-easy+TBM-hard) domains, and 45 template-free (FM+FM/TBM) domains. The list of alignment tools is sorted by the number of times generating the best models. The averaged GDT-TS score of the best models that used the alignments generated by each tool for the three domain sets is calculated and provided in the last three columns. Each alignment tool generates up to 10 structural models from its identified significant templates, and around 150~200 models are generated by all alignment tools. We analyzed the quality of the best models that were generated by all methods, including both alignment methods and *ab initio* modeling methods. The average GDT-TS scores of the best models at 100-point scale for the three domain sets are 59.36 (all domains), 40.75 (FM+FM/TBM) and 71.86 (TBM+TBM-hard) respectively. When calculating the averaged GDT-TS of the best models that used the alignments generated by all alignment tools, the averaged GDT-TS score can reach 57.95 for 112 domains, 37.25 for free-modeling domains, and 71.84 for template-based domains respectively, which are better than the results of the single best alignment tool – HHsuite. The results demonstrate that using multiple alignment tools can generate protein structures with higher quality as a whole than using the single best alignment method. Our tertiary structure predictors (MULTICOM-CONSTRUCT and MULTICOM-CLUSTER didn’t reach the highest possible accuracy if the best models of all domains were selected. This demonstrates that our quality assessment methods can still be improved.

| Data | Contact | Correlation | Loss |
| --- | --- | --- | --- |
| CASP12 | DeepRank-NoContact | 0.832 | 0.054 |
|  | **DeepRank-WithContact** | **0.853** | **0.048** |
|  | Average features | 0.764 | 0.067 |
|  | Average feature_zscore | 0.720 | 0.064 |
| CASP13 (74 targets) | DeepRank-NoContact | 0.869 | 0.059 |
|  | **DeepRank-WithContact** | **0.898** | **0.051** |
|  | Average_feature | 0.079 | 0.073 |
|  | Average_feature_zscore | 0.788 | 0.057 |

**Table S4.** Deep learning and contact prediction improved protein model quality assessment in CASP12 and CASP 13 datasets.

|  | **Loss** | | | **Correlation** | | |
| --- | --- | --- | --- | --- | --- | --- |
| **Method** | **All** | **TBM +**  **TBM-hard** | **FM +**  **FM/TBM** | **All** | **TBM + TBM-hard** | **FM + FM/TBM** |
| **DeepRank**  **No-Contact** | 0.059 | 0.046 | 0.066 | 0.869 | 0.936 | 0.824 |
| **DeepRank with Contacts** | **0.051** | **0.040** | **0.061** | **0.898** | **0.963** | **0.857** |

**Table S5.** Impact of contact features on protein model quality assessment in CASP13 dataset.

| Method | Features | Loss | | | Correlation | | |
| --- | --- | --- | --- | --- | --- | --- | --- |
|  |  | With Scwrl | Without Scwrl | <With> - <Without> | With Scwrl | Without Scwrl | <With> - <Without> |
| Individual QA features used in DeepRank | Pcons | 0.065 | 0.065 | 0.0000 | 0.891 | 0.891 | 0.0000 |
|  | APOLLO | 0.068 | 0.068 | 0.0000 | 0.904 | 0.904 | 0.0000 |
|  | ModFoldclust2 | 0.069 | 0.069 | 0.0000 | 0.883 | 0.883 | 0.0000 |
|  | SBROD | 0.086 | 0.087 | -0.0004 | 0.711 | 0.711 | 0.0000 |
|  | ProQ2 | 0.101 | 0.223 | -0.1222 | 0.724 | 0.577 | 0.1467 |
|  | RWplus | 0.102 | 0.096 | 0.0063 | 0.639 | 0.590 | 0.0492 |
|  | ProQ3 | 0.102 | 0.111 | -0.0082 | 0.689 | 0.438 | 0.2509 |
|  | Dope | 0.107 | 0.091 | 0.0163 | 0.617 | 0.586 | 0.0307 |
|  | ProQ3_highres | 0.109 | 0.108 | 0.0012 | 0.677 | 0.186 | 0.4908 |
|  | DeepQA | 0.109 | 0.110 | -0.0007 | 0.666 | 0.668 | -0.0015 |
|  | ProQ3_lowres | 0.110 | 0.096 | 0.0141 | 0.698 | 0.248 | 0.4504 |
|  | Voronota | 0.112 | 0.100 | 0.0121 | 0.725 | 0.711 | 0.0150 |
|  | OPUS-PSP | 0.120 | 0.121 | -0.0007 | 0.537 | 0.459 | 0.0782 |
|  | DNCON2_medium-range | 0.149 | 0.154 | -0.0050 | 0.674 | 0.665 | 0.0090 |
|  | DNCON2_short-range | 0.151 | 0.150 | 0.0014 | 0.658 | 0.647 | 0.0104 |
|  | Solvent | 0.160 | 0.163 | -0.0036 | 0.634 | 0.604 | 0.0300 |
|  | RF_SRS | 0.168 | 0.164 | 0.0041 | 0.526 | 0.531 | -0.0052 |
|  | SS_penalty | 0.172 | 0.172 | 0.0000 | 0.429 | 0.429 | 0.0000 |
|  | SS_similarity | 0.175 | 0.175 | 0.0000 | 0.464 | 0.464 | 0.0000 |
|  | DNCON2_long-range | 0.183 | 0.180 | 0.0031 | 0.654 | 0.645 | 0.0095 |
|  | Weighted_exposed | 0.241 | 0.200 | 0.0411 | 0.496 | 0.502 | -0.0066 |
|  | Total_surf_area | 0.261 | 0.213 | 0.0484 | 0.487 | 0.492 | -0.0054 |
|  | EuclideanPairDist | 0.303 | 0.303 | 0.0000 | 0.375 | 0.375 | 0.0000 |
| Feature Ensemble | Average_feaure | 0.074 | 0.068 | 0.0056 | 0.792 | 0.477 | 0.3147 |
|  | Average_feaure_zscore | 0.058 | 0.071 | -0.0131 | 0.787 | 0.770 | 0.0175 |
| DeepRank 10-fold predictions | DeepRank_fold1 | 0.056 | 0.058 | -0.0025 | 0.884 | 0.876 | 0.0080 |
|  | DeepRank_fold2 | 0.054 | 0.057 | -0.0036 | 0.886 | 0.874 | 0.0118 |
|  | DeepRank_fold3 | 0.056 | 0.056 | 0.0005 | 0.882 | 0.880 | 0.0024 |
|  | DeepRank_fold4 | 0.054 | 0.059 | -0.0052 | 0.885 | 0.883 | 0.0012 |
|  | DeepRank_fold5 | 0.055 | 0.057 | -0.0024 | 0.884 | 0.872 | 0.0120 |
|  | DeepRank_fold6 | 0.056 | 0.080 | -0.0243 | 0.886 | 0.853 | 0.0329 |
|  | DeepRank_fold7 | 0.058 | 0.058 | -0.0001 | 0.881 | 0.879 | 0.0024 |
|  | DeepRank_fold8 | 0.058 | 0.068 | -0.0096 | 0.882 | 0.867 | 0.0152 |
|  | DeepRank_fold9 | 0.055 | 0.057 | -0.0028 | 0.887 | 0.846 | 0.0400 |
|  | DeepRank_fold10 | 0.059 | 0.058 | 0.0009 | 0.878 | 0.849 | 0.0287 |
| DeepRank Ensemble | DeepRank_10fold_average | 0.054 | 0.058 | -0.0033 | 0.884 | 0.880 | 0.0045 |
|  | DeepRank | 0.052 | 0.114 | -0.0620 | 0.886 | 0.837 | 0.0492 |

**Table S6**. The effects of the model side-chain repacking on the performance of DeepRank, averaging methods, and individual quality assessment (QA) features. The following four ensemble techniques are also evaluated: (1) Average_feature: averaging all scores of individual features. (2) Average_feature_zscore: averaging the z-scores of individual features. (3) DeepRank_10-fold_average: averaging the 10 predicted scores from 10 DeepRank networks which were trained using 10-fold cross-validation. (4) DeepRank: Two-level neural network quality assessment method, which feeds the 10 predicted scores and all individual features into another one network to predict one quality score. To fairly compare the performance of model quality assessment methods with or without side-chain repacking, we re-run DeepRank on the two sets of server models of 74 targets (i.e., with or without side-chain repacking by SCWRL program). The models used for this analysis are the same set used in MULITCOM Human predictor, in which the incomplete models whose amino acid sequences in structures did not match their original sequences or the highly similar models (e.g., GDT-TS > 0.95) from the same server group were filtered out. The per-target comparison of DeepRank with or without side-chain repacking is visualized in **Figure S11**.

| Predictor | # | GDT-TS | | | TM-score | | | RMSD | | |
| --- | --- | --- | --- | --- | --- | --- | --- | --- | --- | --- |
|  |  | Before | After | P-value | Before | After | P-value | Before | After | P-value |
| MULTICOM CLUSTER | 62 | 0.45 | **0.56** | 8.8E-06 | 0.50 | **0.62** | 3.6E-06 | 16.08 | **8.99** | 2.4E-05 |
| MULTICOM CONSTRUCT | 62 | 0.45 | **0.55** | 9.5E-06 | 0.50 | **0.60** | 7.6E-06 | 30.38 | **8.78** | 4.7E-06 |
| MULTICOM (HUMAN) | 48 | 0.64 | **0.66** | 7.8E-03 | 0.67 | **0.69** | 2.3E-02 | 7.75 | **5.93** | 7.7E-02 |

**Table S7**. Effect of domain parsing on the protein structure prediction. The protein targets which were treated as multi-domain proteins in CASP13 by MULTICOM predictors are evaluated.  MULTICOM server predictor predicted 31 out of 90 targets as multi-domain proteins, while MULTICOM human predictor modeled 19 targets as multi-domain ones. The second column lists the total number of individual domains of the multi-domain targets. The results show that the quality after domain parsing is always better than not using domain parsing for the three predictors.

| **Target** | Classification | Loss | **Target** | Classification | Loss | **Target** | Classification | Loss |
| --- | --- | --- | --- | --- | --- | --- | --- | --- |
| **T0953s2** | FM/TBM+FM | 0.14 | **T0980s2** | FM | 0.11 | **T1019s1** | FM/TBM | 0.14 |
| **T0957s1** | FM+TBM-hard | 0.12 | **T0991** | FM | 0.12 | **T1022s2** | TBM-hard | 0.1 |
| **T0968s1** | FM | 0.16 | **T0992** | FM/TBM | 0.13 |  |  |  |
| **T0975** | FM | 0.2 | **T0998** | FM | 0.16 |  |  |  |
| **T0978** | FM/TBM | 0.14 | **T1008** | FM/TBM | 0.2 |  |  |  |
| **T0979** | TBM-hard | 0.12 | **T1010** | FM | 0.3 |  |  |  |

**Table S8.** CASP13 targets with model selection loss > 0.1 by DeepRank quality assessment (QA) method.

| Rank | GR Code | GR name | Count | Avg. GDT-TS | Sum Zscore  (>0.0) | Avg. RMSD | Avg. LDDT | Avg. TM-score |
| --- | --- | --- | --- | --- | --- | --- | --- | --- |
| 1 | 145 | QUARK | 112 | 62.37 | 98.83 | 3.1 | 0.56 | 0.68 |
| 2 | 261 | Zhang-Server | 112 | 62.48 | 97.17 | 3 | 0.57 | 0.68 |
| 3 | 324 | RaptorX-DeepModeller | 112 | 61.19 | 87.57 | 3.41 | 0.56 | 0.67 |
| 4 | 221 | RaptorX-TBM | 112 | 58.66 | 65.99 | 3.35 | 0.54 | 0.64 |
| 5 | 368 | BAKER-ROSETTASERVER | 111 | 55.24 | 51.45 | 2.99 | 0.52 | 0.61 |
| 6 | 498 | RaptorX-Contact | 112 | 53.26 | 46.33 | 4.32 | 0.5 | 0.6 |
| **7** | **243** | **MULTICOM-CONSTRUCT** | **112** | **54.6** | **37.2** | **3.04** | **0.5** | **0.6** |
| **8** | **058** | **MULTICOM_CLUSTER** | **112** | **53.81** | **34.8** | **3.29** | **0.49** | **0.59** |
| **9** | **023** | **MULTICOM-NOVEL** | **112** | **53.79** | **32.26** | **3.5** | **0.5** | **0.59** |
| 10 | 164 | Yang-Server | 109 | 53.4 | 28.78 | 3.02 | 0.46 | 0.59 |

**Table S9.** The official CASP13 evaluation of top 10 server predictors on all 112 “all groups + server only” domains. Predictors were ranked by the sum of Z-score of the first (i.e. TS1) submitted models predicted by all predictors. Z-scores were calculated according to the GDT-TS scores of predicted models. The results of three MULTICOM server predictors (MULTICOM_CLUSTER, MULTICOM-CONSTRUCT and MULTICOM-NOVEL) are highlighted in the table. All the results are compiled from the official data released at the CASP13 website. These 10 predictors came from five research labs: Zhang Lab (QUARK and Zhang-Server), Xu Lab (RaptorX-DeepModeller, RaptorX-TBM, RaptorX-Contact), Baker Lab (ROSETTASERVER), Cheng Lab (MULTICOM-CONSTRUCT, MULTICOM_CLUSTER, MULTICOM-NOVEL) and Yang Lab (Yang-Server).

**Figures**

**Section I. Performance of MULTICOM structure prediction methods**

**
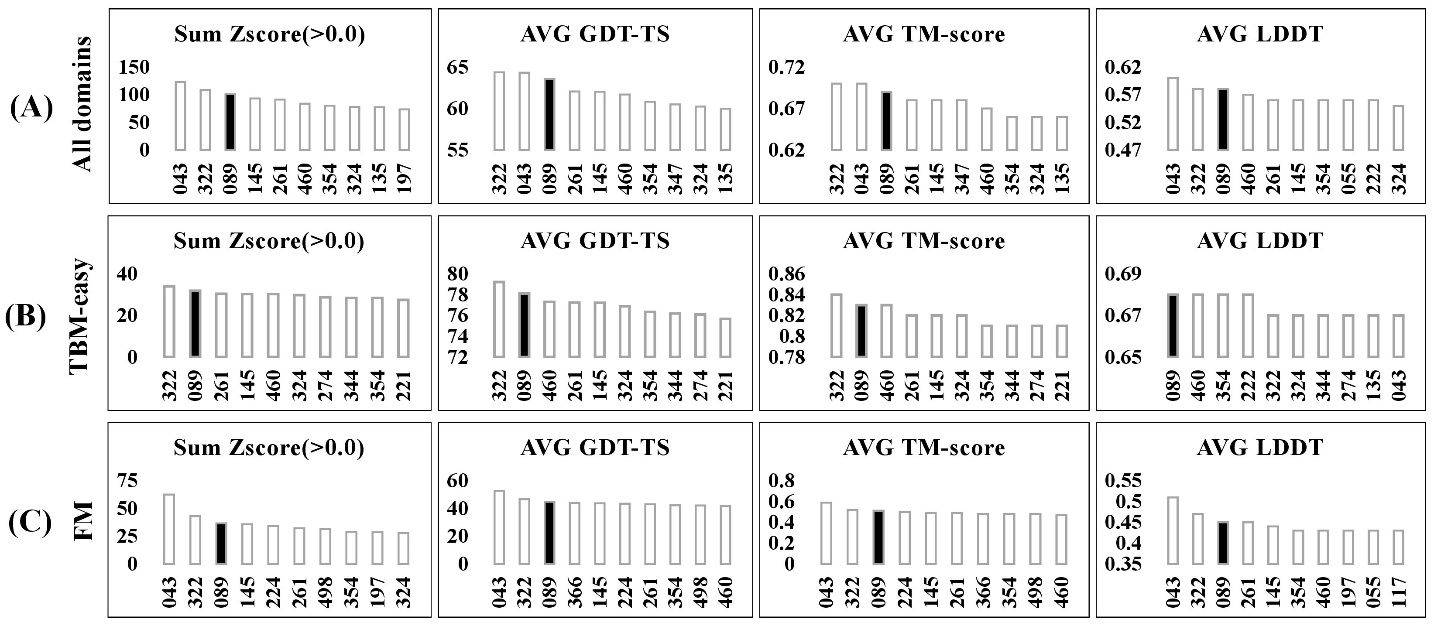
**

**Figure S1.** The official CASP13 evaluation of top 10 predictors out of 98 human and server predictors on 104 “all groups” targets. Predictors are ranked based on four different metrics of the first (i.e. TS1) submitted models predicted by all predictors. All the results are compiled from the official data released at the CASP13 website. (A) Evaluation on 104 domains. (B) Evaluation on 40 template-based (TBM-easy) domains. (C) Evaluation on 31 template-free (FM) domains. The bar highlighted in black is the performance of MULTICOM human predictor (group number: ‘089’) in CASP13. The figures were drawn according to the results compiled from the official data released at the CASP13 website.


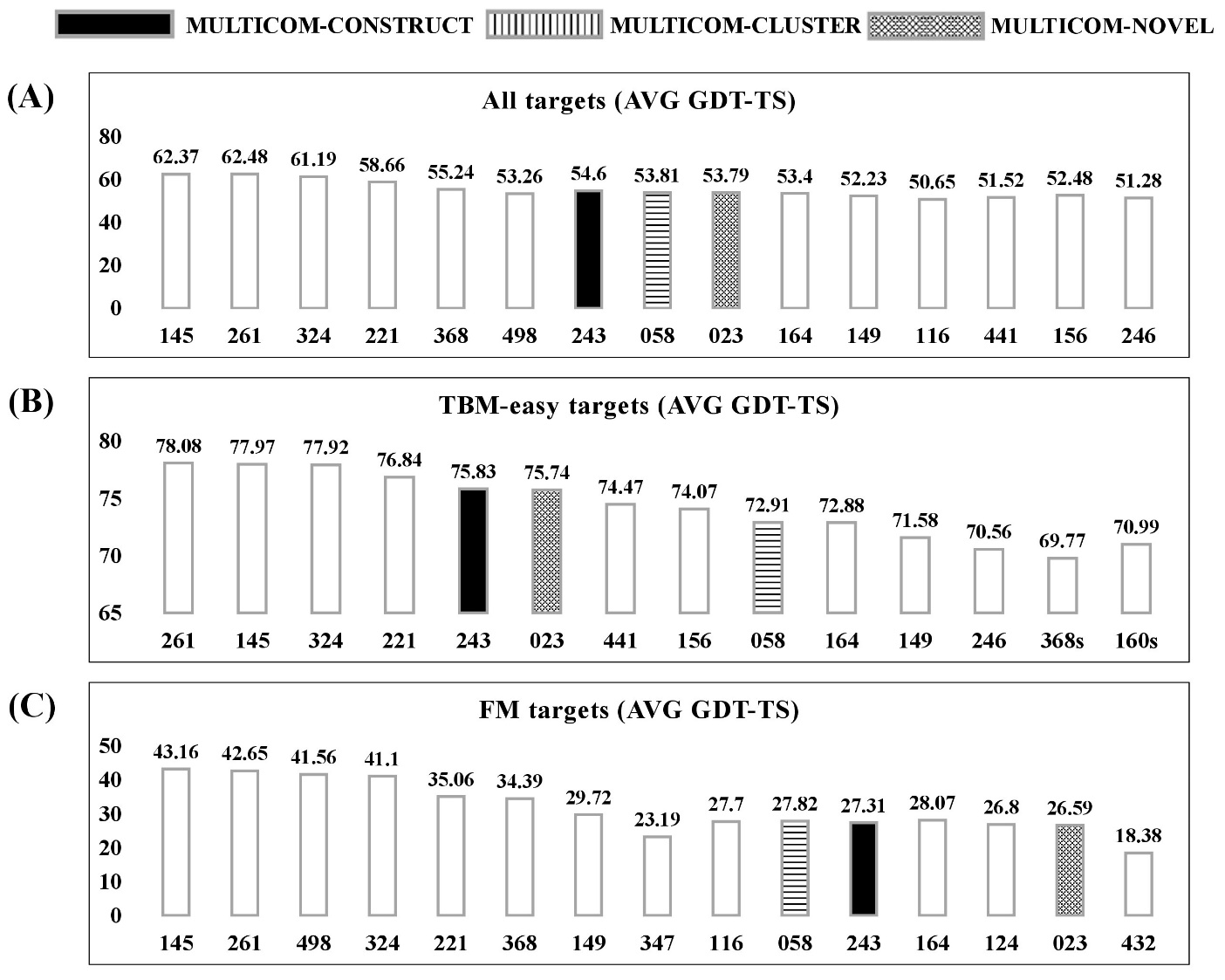


**Figure S2.** The official CASP13 evaluation of top 15 server predictors on 112 “all groups + server only” domains. Predictors are ranked by sum of Z-score of the first (i.e. TS1) submitted models predicted by all predictors denoted by each bar. Z-score (not shown) of each predictor was calculated according to GDT-TS scores of predicted models. The average GDT-TS score at the 100-point scale of each predictor is shown at the top of each bar and the server group code at the bottom. All the results are compiled from the official data released at the CASP13 website. (A) Evaluation on 112 domains. (B) Evaluation on 45 template-based (TBM-easy) domains. (C) Evaluation on 32 template-free (FM) domains.  The highlighted bars are the performance of MULTICOM-CONSTRUCT (black filled), MULTICOM-CLUSTER (Horizontal stripes), MULTICOM-NOVEL (Dotted) in CASP13. The detailed results are summarized in **Table S9**.

**
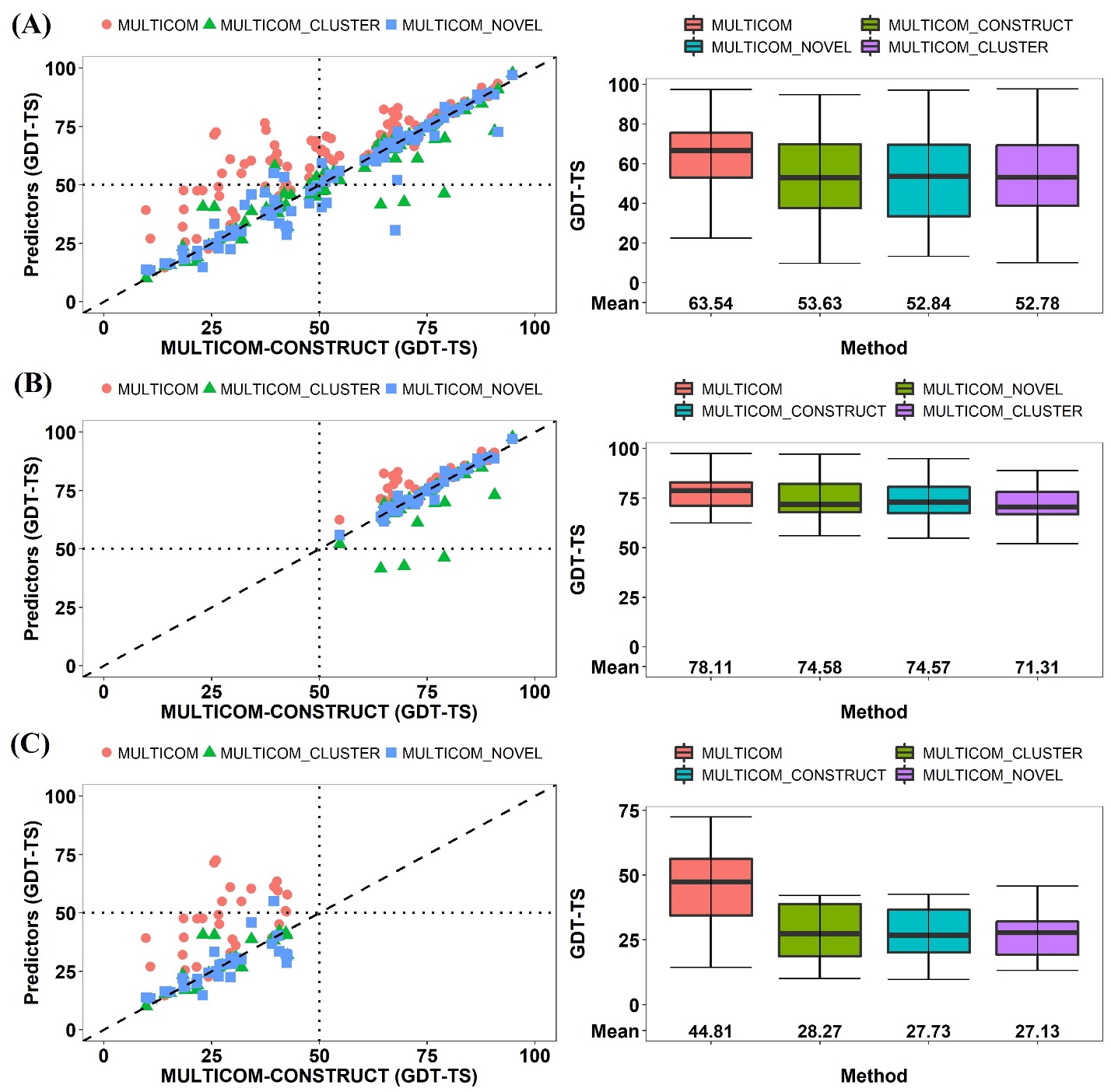
**

**Figure S3.** Evaluation of four MULTICOM predictors by GDT-TS score. The methods are ranked by average GDT-TS score of the first (i.e. TS1) submitted models. **(A)** on 104 domains (Left plot: GDT-TS scores of MULTICOM, MULTICOM_CLUSTER, MULTICOM-NOVEL models versus GDT-TS scores of MULTICOM-CONSTRUCT models; Right plot: mean and variation of the GDT-TS scores of the models of the four methods). **(B)** on 40 template-based (TBM-easy) domains. **(C)** on 31 template-free (FM) domains.

**
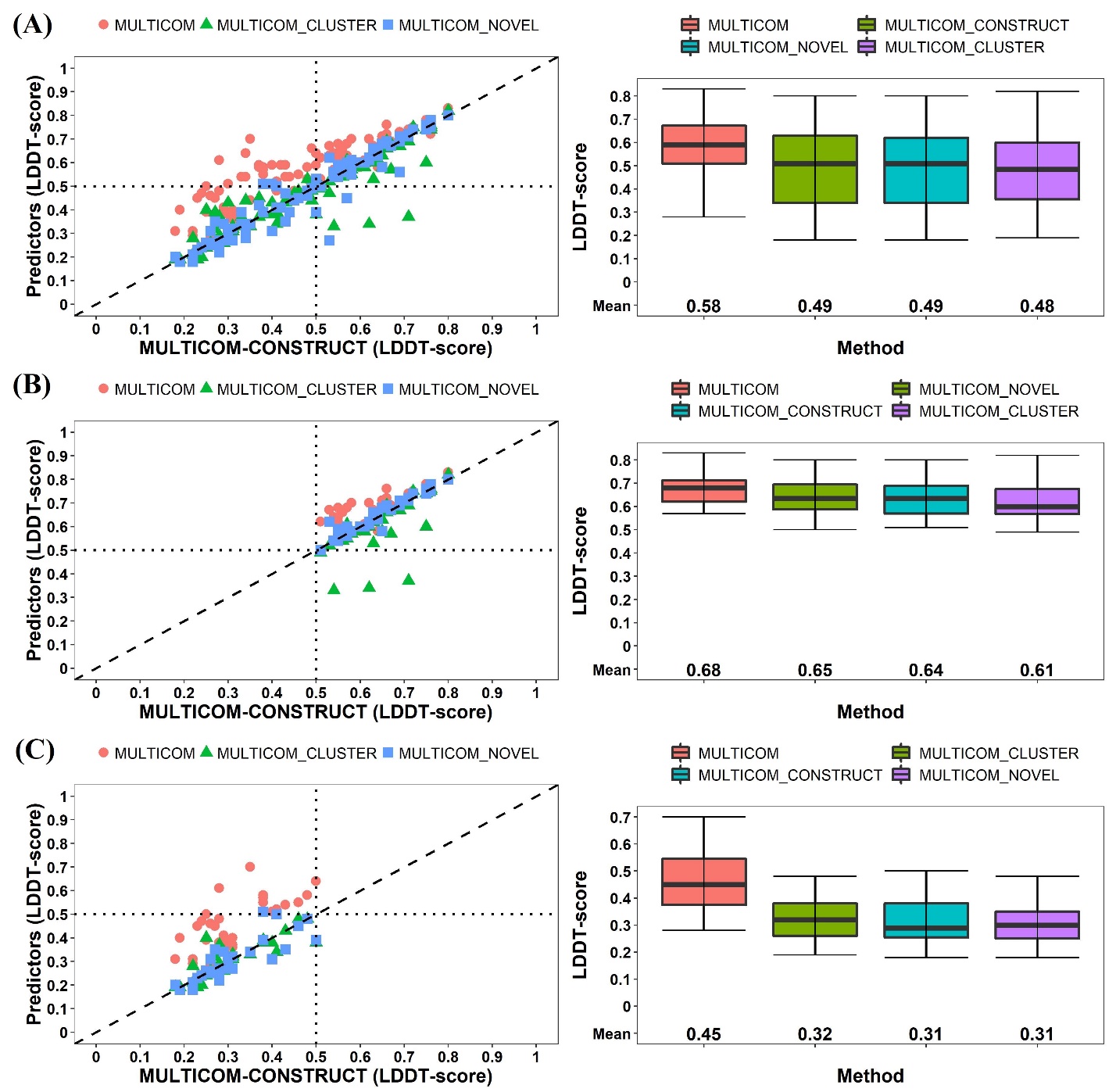
**

**Figure S4.** Evaluation of four MULTICOM predictors by LDDT score. The methods are ranked by average LDDT score of the first (i.e. TS1) submitted models. **(A)** on 104 domains (Left plot: LDDT scores of MULTICOM, MULTICOM_CLUSTER, MULTICOM-NOVEL models versus LDDT scores of MULTICOM-CONSTRUCT models; Right plot: mean and variation of the LDDT scores of the models of the four methods). **(B)** on 40 template-based (TBM-easy) domains. **(C)** on 31 template-free (FM) domains.

**Section II. Performance of DeepRank for model selection**

**
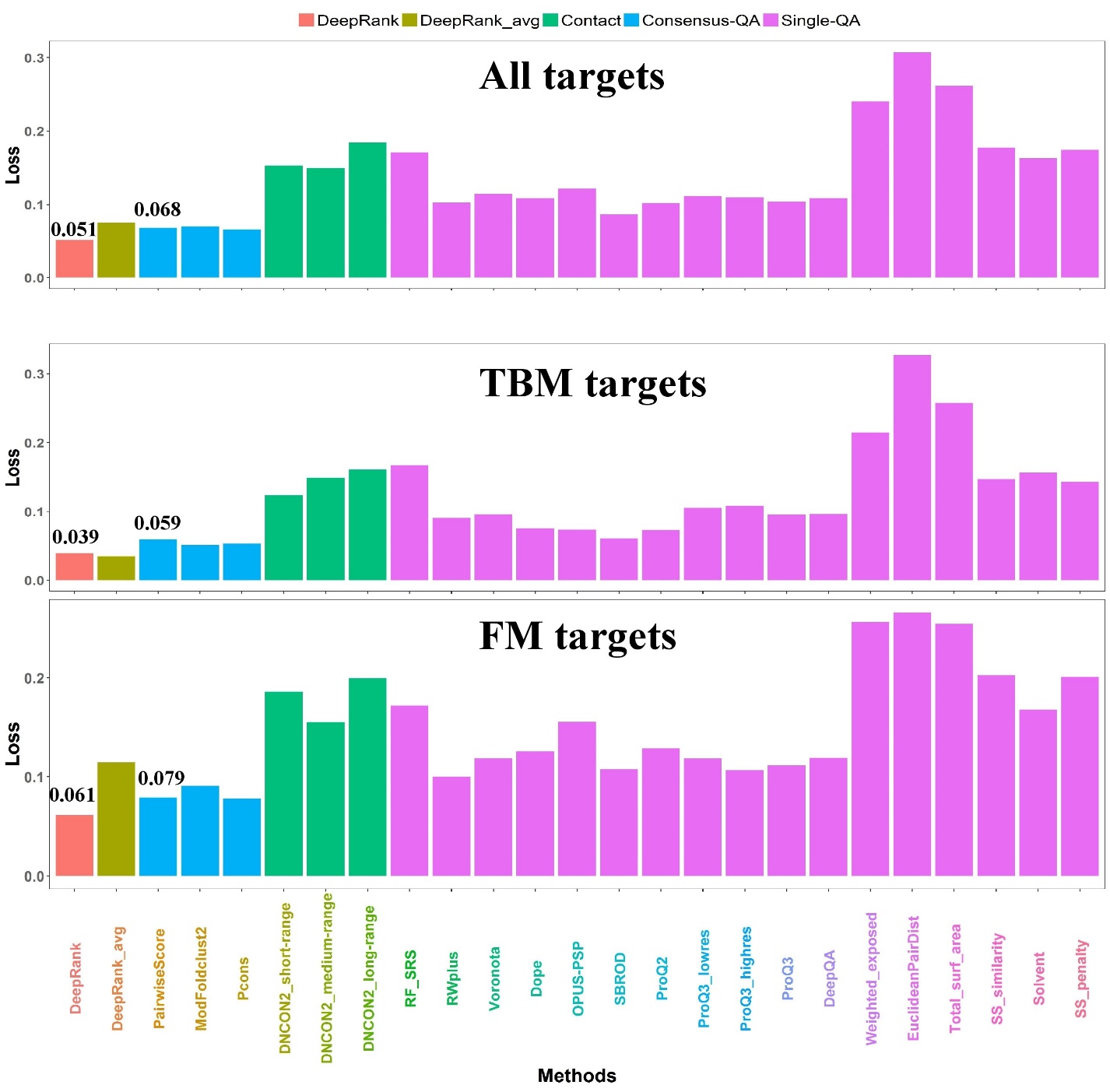
**

**Figure S5.** Comparison of DeepRank with individual QA features on CASP13 targets. The methods are evaluated according to average GDT-TS loss calculated from the 74 full-length targets, 40 templated-based (TBM-easy and TBM-hard) targets, and 37 free-modeling targets (FM+FM/TBM), respectively.


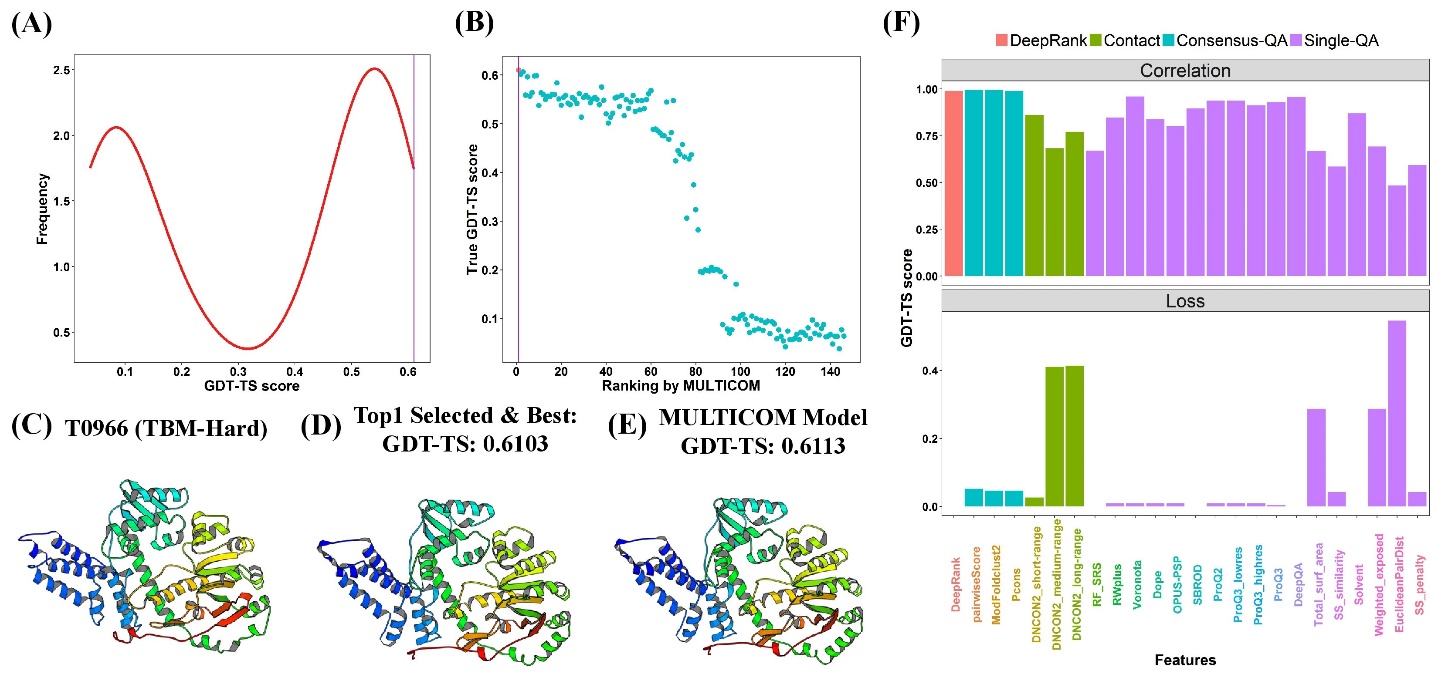


**Figure S6.** Tertiary structure prediction for T0966. **(A)** The distribution of GDT-TS scores of 146 server models. **(B)** The plot of the true GDT-TS scores of models against their predicted ranking by MULTICOM. The point highlighted in red is the top model selected by DeepRank. **(C)** The native structure of target T0966 (PDB code: [5w6l](http://www.rcsb.org/pdb/cgi/explore.cgi?pdbId=5w6l)). **(D)** The top selected model. **(E)** The final first MULTICOM model (TS1). **(F)** The ranking of individual QA methods for target T0966.


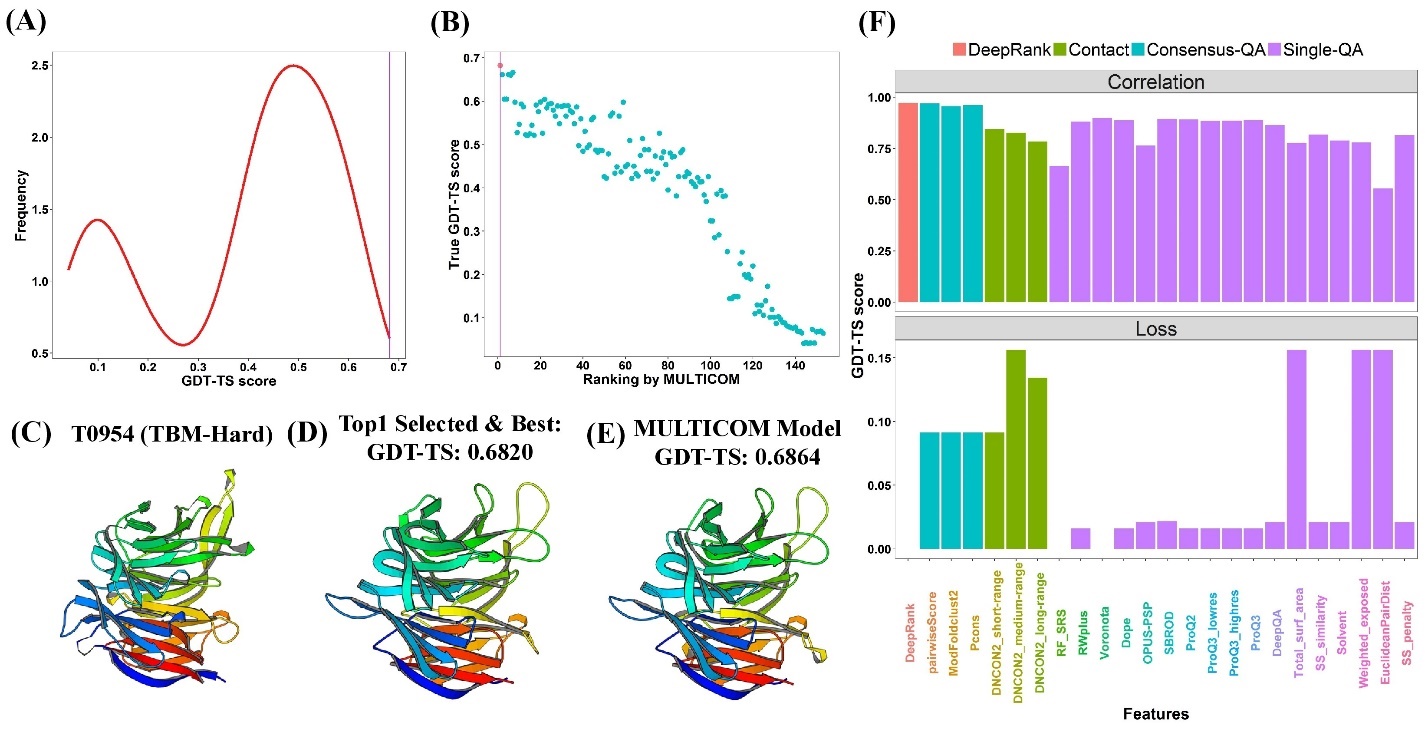


**Figure S7.** Tertiary structure prediction for T0954. **(A)** The distribution of GDT-TS scores of 153 server models. **(B)** The plot of the true GDT-TS scores of models against their predicted ranking by MULTICOM. The point highlighted in red is the top model selected by DeepRank. **(C)** The native structure of target T0954 (PDB code: [6cvz](http://www.rcsb.org/pdb/cgi/explore.cgi?pdbId=6cvz)). **(D)** The top selected model. **(E)** The final first MULTICOM model (TS1). **(F)** The ranking of individual QA methods for target T0954.


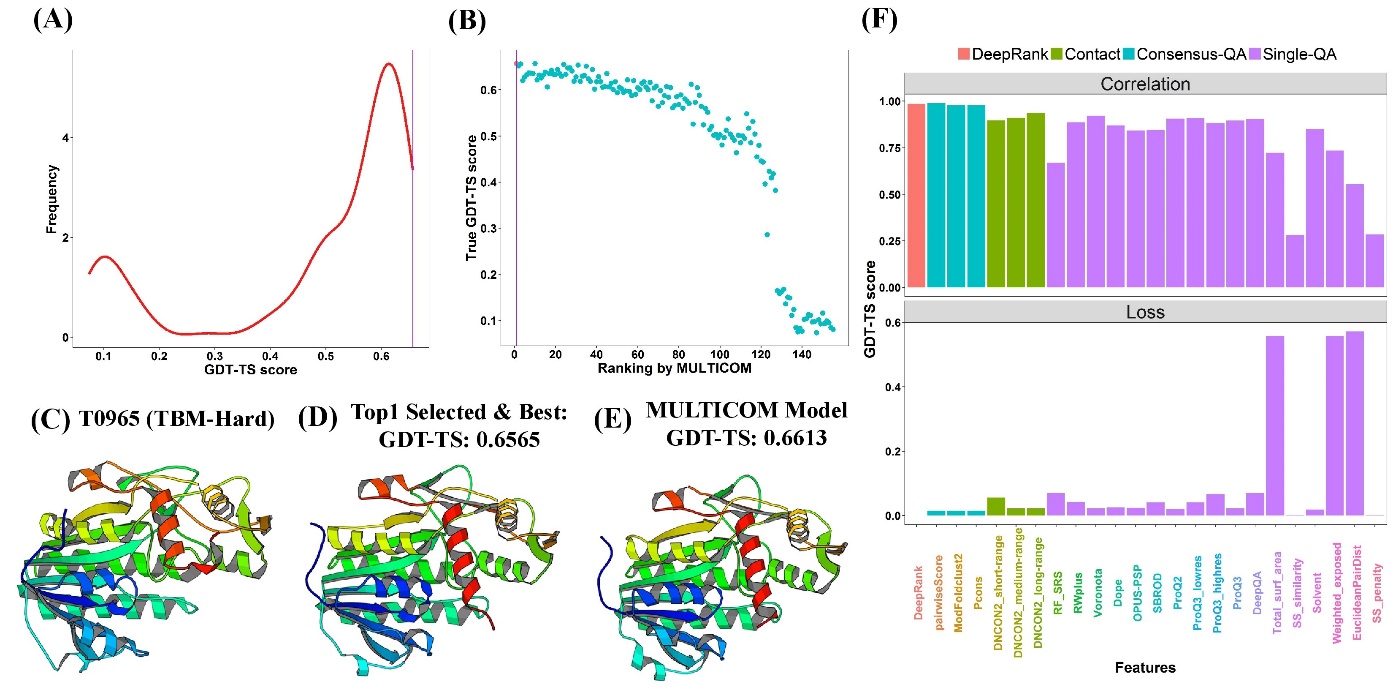


**Figure S8.** Tertiary structure prediction for T0965. **(A)** The distribution of GDT-TS scores of 155 server models. **(B)** The plot of the true GDT-TS scores of models against their predicted ranking by MULTICOM. The point highlighted in red is the top model selected by DeepRank. **(C)** The native structure of target T0965 (PDB code: [6d2v](http://www.rcsb.org/pdb/cgi/explore.cgi?pdbId=6d2v)). **(D)** The top selected model. **(E)** The final first MULTICOM model (TS1)**. (F)** The ranking of individual QA methods for target T0965.


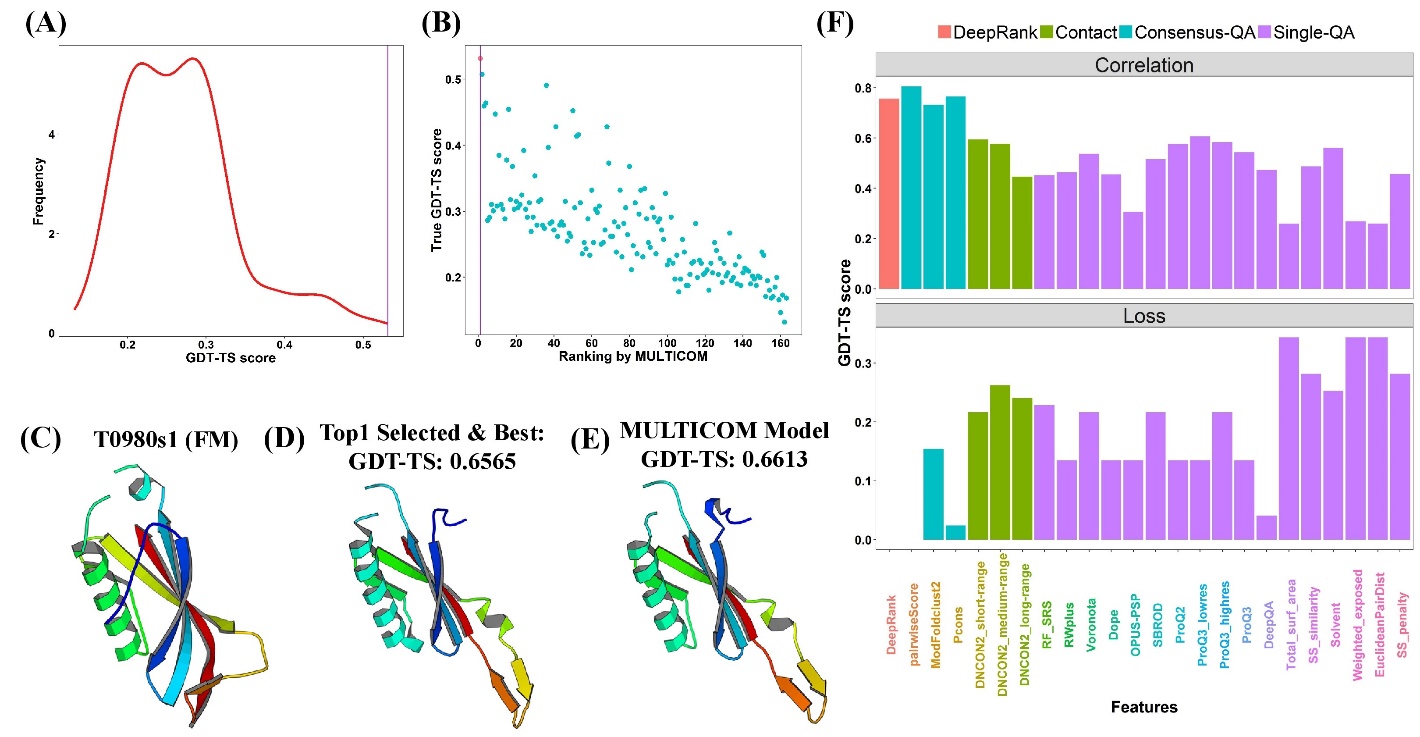


**Figure S9.** Tertiary structure prediction for T0980s1. **(A)** The distribution of GDT-TS scores of 163 server models. **(B)** The plot of the true GDT-TS scores of models against their predicted ranking by MULTICOM. The point highlighted in red is the top model selected by DeepRank. **(C)** The native structure of target T0980s1 (PDB code: [6gnx](http://www.rcsb.org/pdb/cgi/explore.cgi?pdbId=6gnx)). **(D)** The top selected model. **(E)** The final first MULTICOM model (TS1). **(F)** The ranking of individual QA methods for target T0980s1.


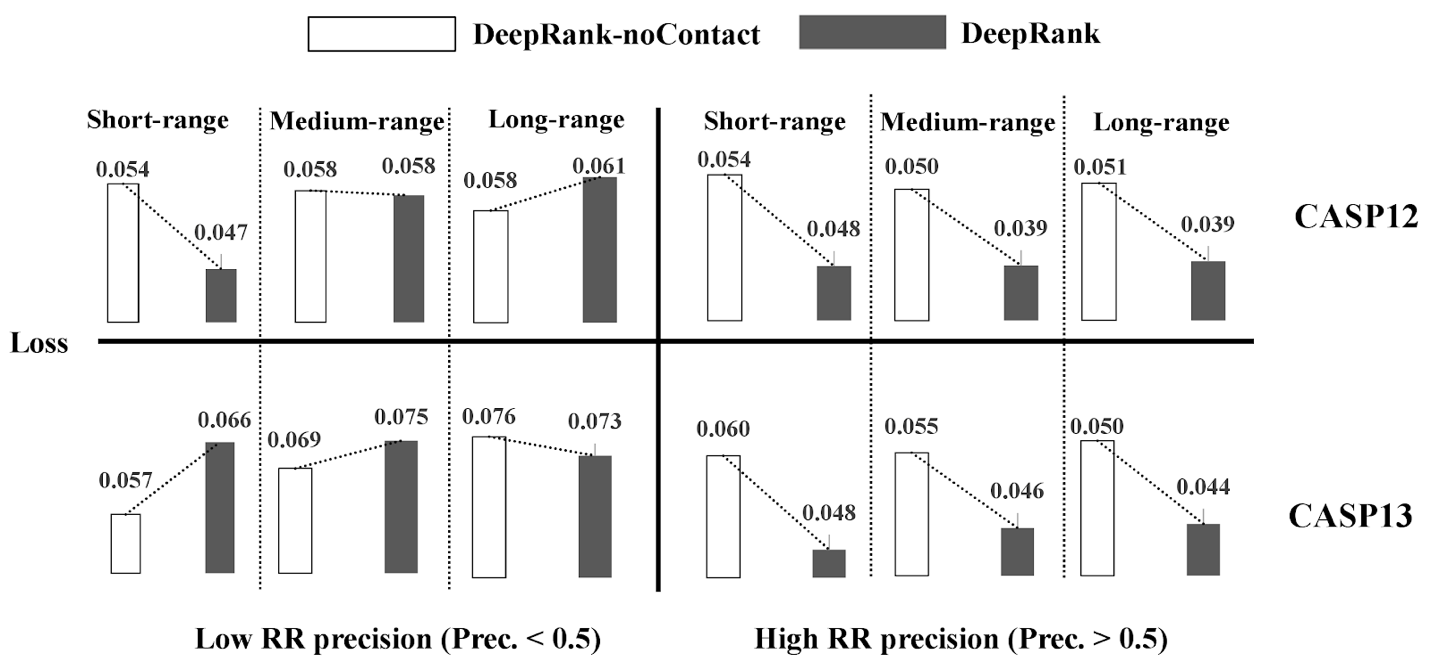


**Figure S10**. Impact of contact prediction accuracy on protein model quality assessment in CASP12 and CASP13 datasets. The white bar represents the loss of DeepRank QA method without contact information, while the black bar represents that of the DeepRank method with contact information. The loss with/without each kind of contact features (short-range, medium-range, long-range) is shown. The loss was consistently reduced on the two datasets if the precision of contacts used with DeepRank is higher than 0.5, otherwise the impact of contacts is mixed.


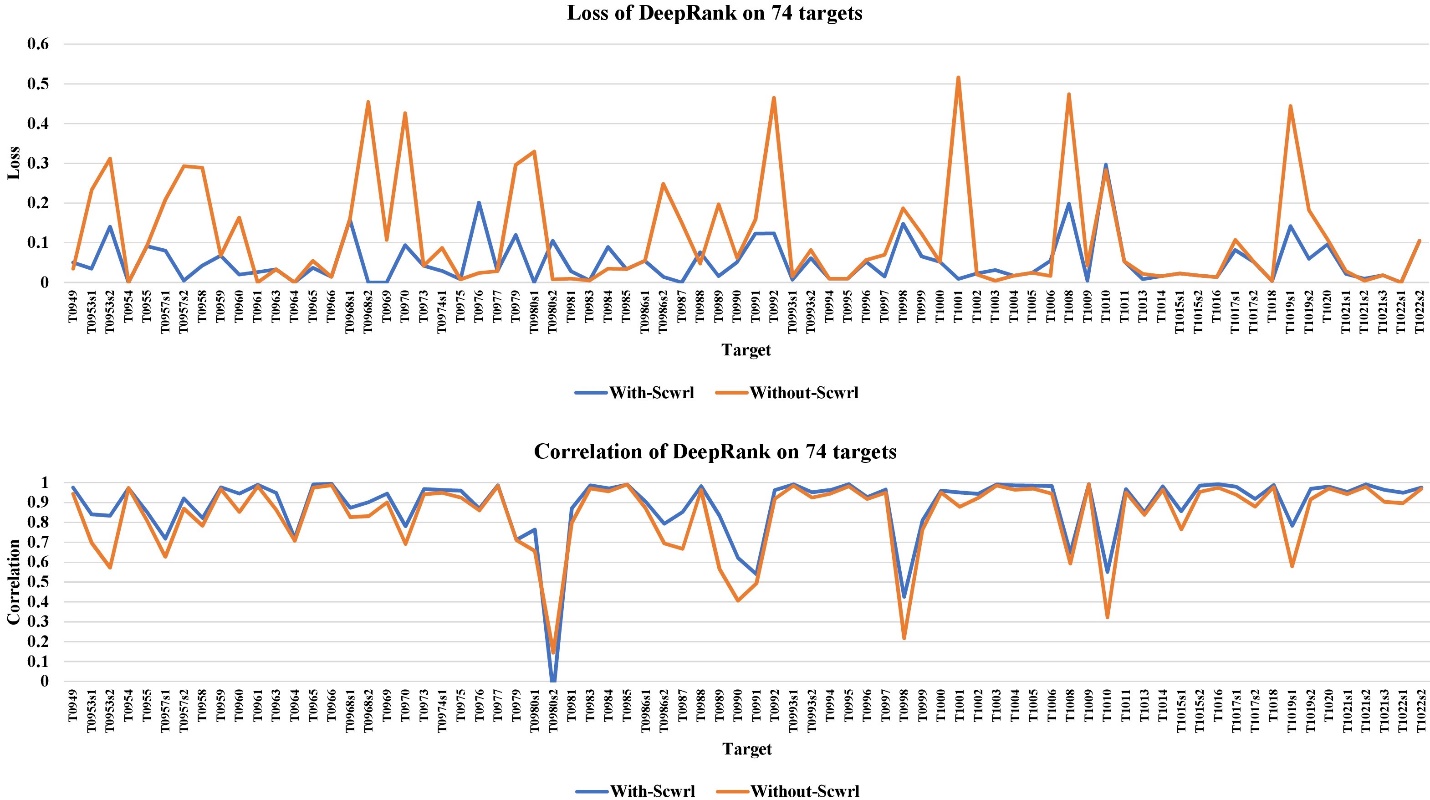


**Figure S11.** Per-target comparison of DeepRank with or without side-chain repacking in terms of loss and correlation. The average loss and correlation for the comparison are summarized in **Table S6**. On 62 out of 74 targets, DeepRank can achieve lower or equal loss using side-chain repacking before evaluating models.


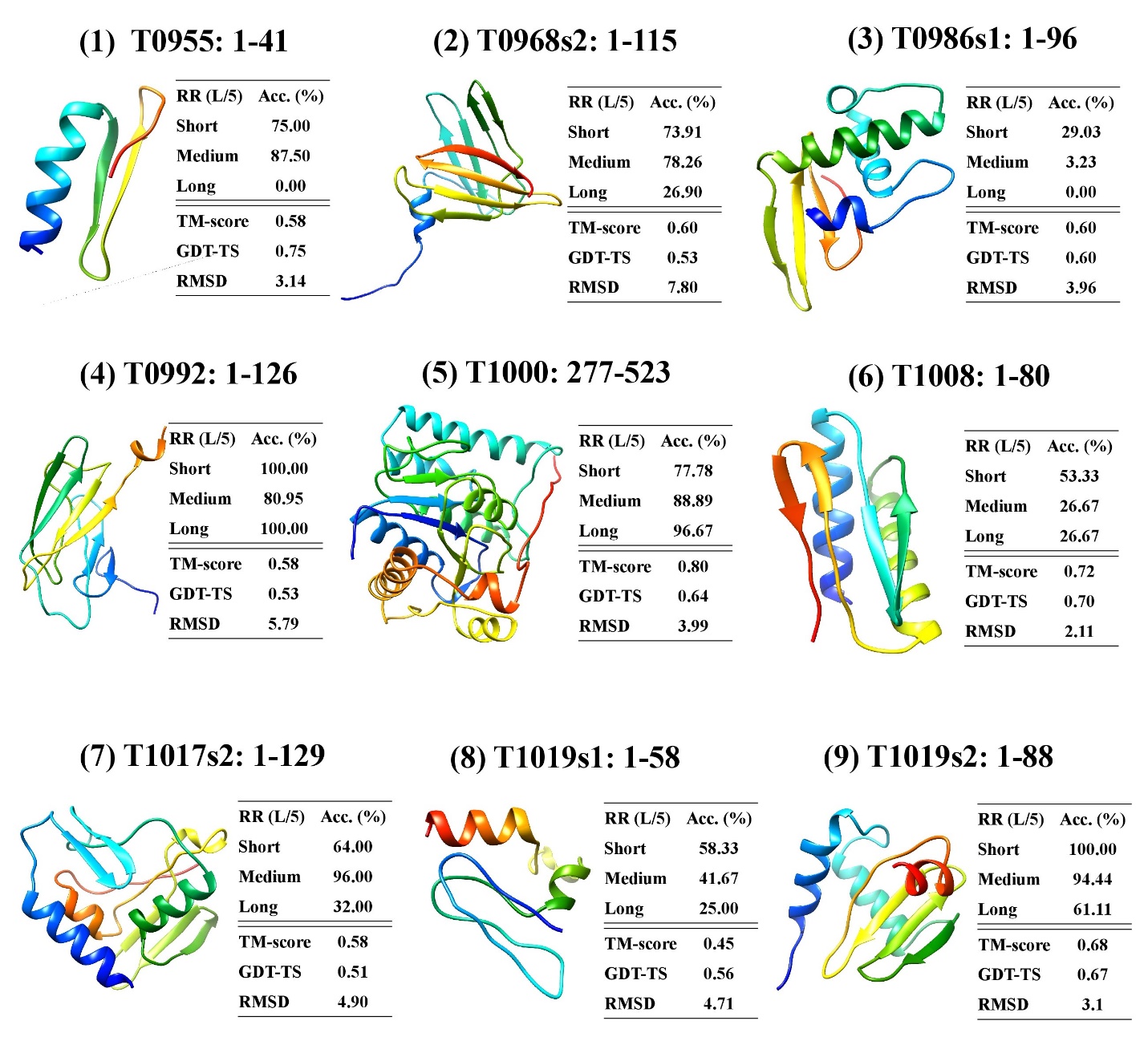


**Figure S12.** Examples of structural models built from predicted contacts for nine hard targets. The region that was modeled using contacts is visualized for each target. The accuracy of predicted top L/5 contacts of short-range, medium-range and long-range is reported in the table next to each model. The quality scores of each model are also provided. The experimental structures of these targets are not officially released and therefore not shown.


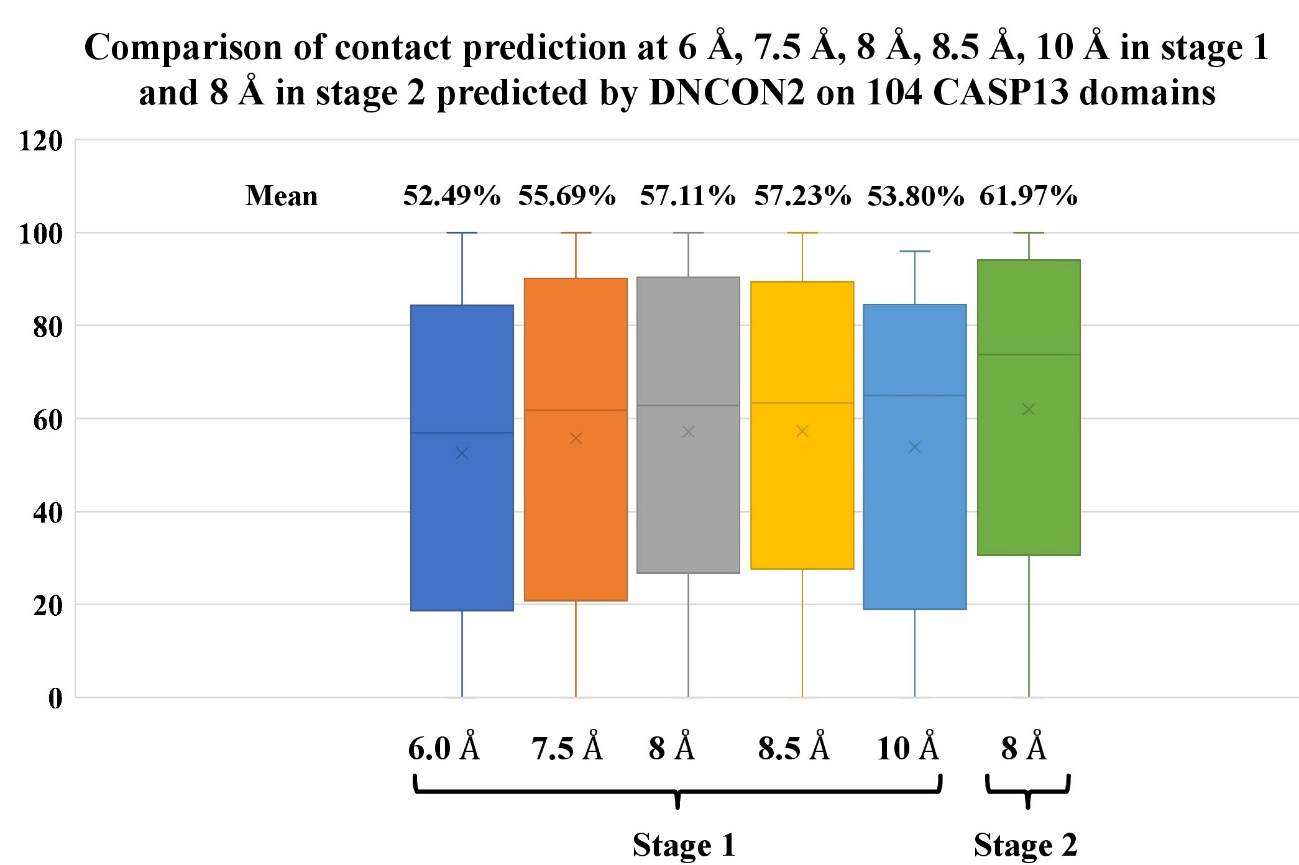


**Figure S13.** Comparison of contact prediction at 6 Å, 7.5 Å, 8 Å, 8.5 Å, 10 Å in stage 1 and 8 Å in stage 2 predicted by DNCON2. The accuracy of top L/5 predicted contact on two stages for 104 CASP13 “all groups” domains was analyzed and compared.


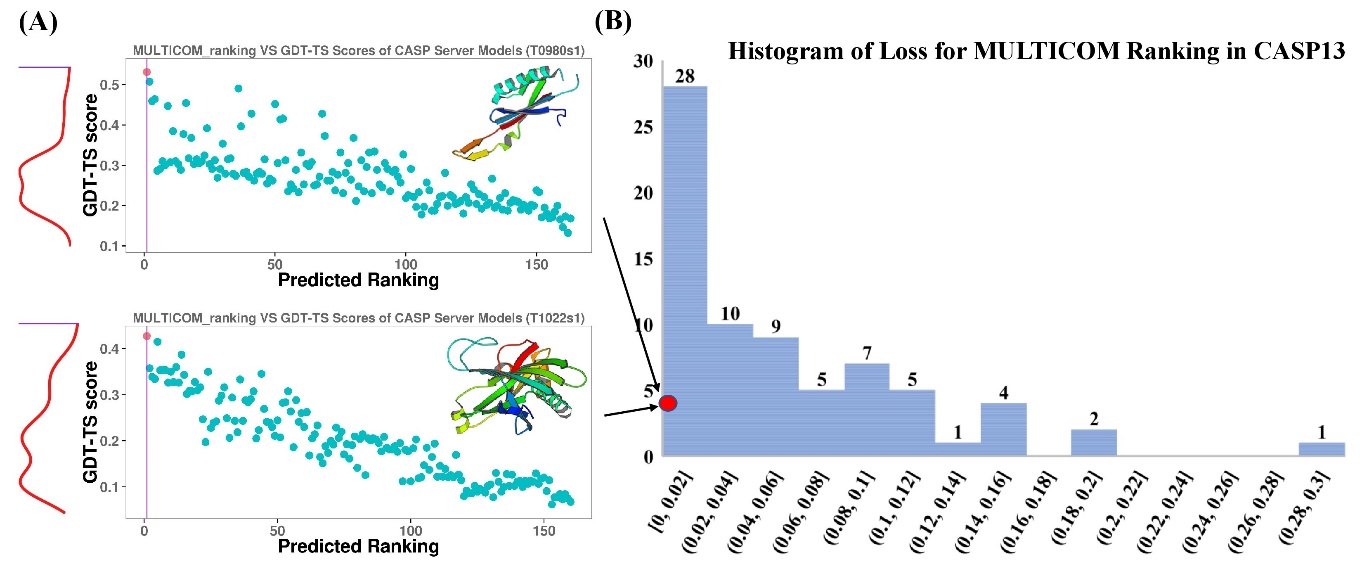


**Figure S14.** Performance of DeepRank in CASP13. (A) Two good examples (T0980s1 and T1022s1) for which MULTICOM selected best server models (red dots) from the model pool. (B) Histogram of loss for DeepRank on CASP13 server models of all the targets. The bin size of loss is set to 0.02. The number of targets for each loss bin is show at the top.


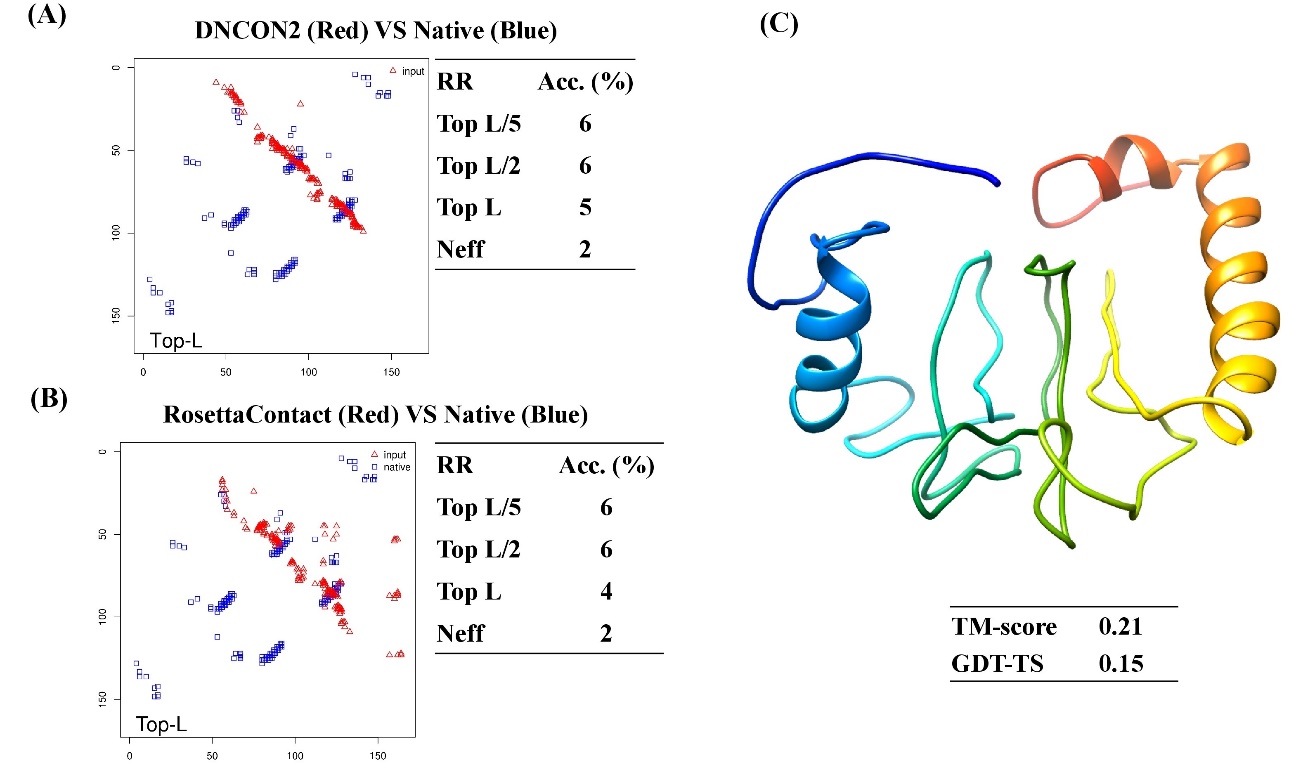


**Figure S15**. Failure of predicting and using contacts in modeling for target T0998. (A) Predicted contact map (red) versus true map (blue), accuracy of top L/5, L/2 and L predicted contacts, number of effective sequences (Neff =2). (B) The contact map of the model built by Rosetta with contacts (red) versus true contact map (blue) and accuracy of the contacts in the model. (C) The predicted structure using RosettaContact and the quality of the model. The contact prediction accuracy is low and the quality of the model is poor. The experimental structure of this target is not officially released and therefore not shown.

**Section III. Failed cases for DeepRank in MULITCOM**


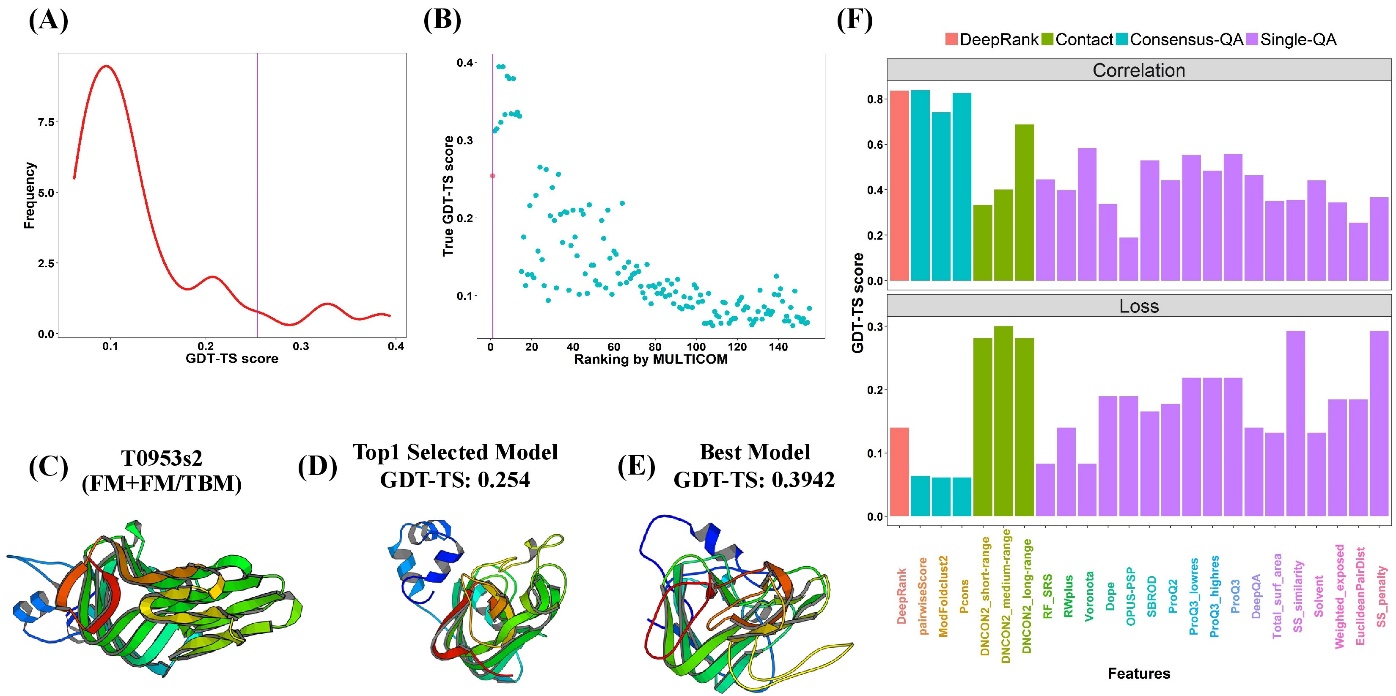


**Figure S16.** DeepRank model quality assessment for T0953s2. **(A)** The distribution of GDT-TS scores of 155 server models. **(B)** The plot of the true GDT-TS scores of models against their predicted ranking by MULTICOM. The point highlighted in red is the top model selected by DeepRank. **(C)** The native structure of target T0953s2 (PDB code: [6f45](http://www.rcsb.org/pdb/cgi/explore.cgi?pdbId=6f45)). **(D)** The top selected model. **(E)** The best server model. **(F)** The ranking of individual QA methods for target T0953s2.


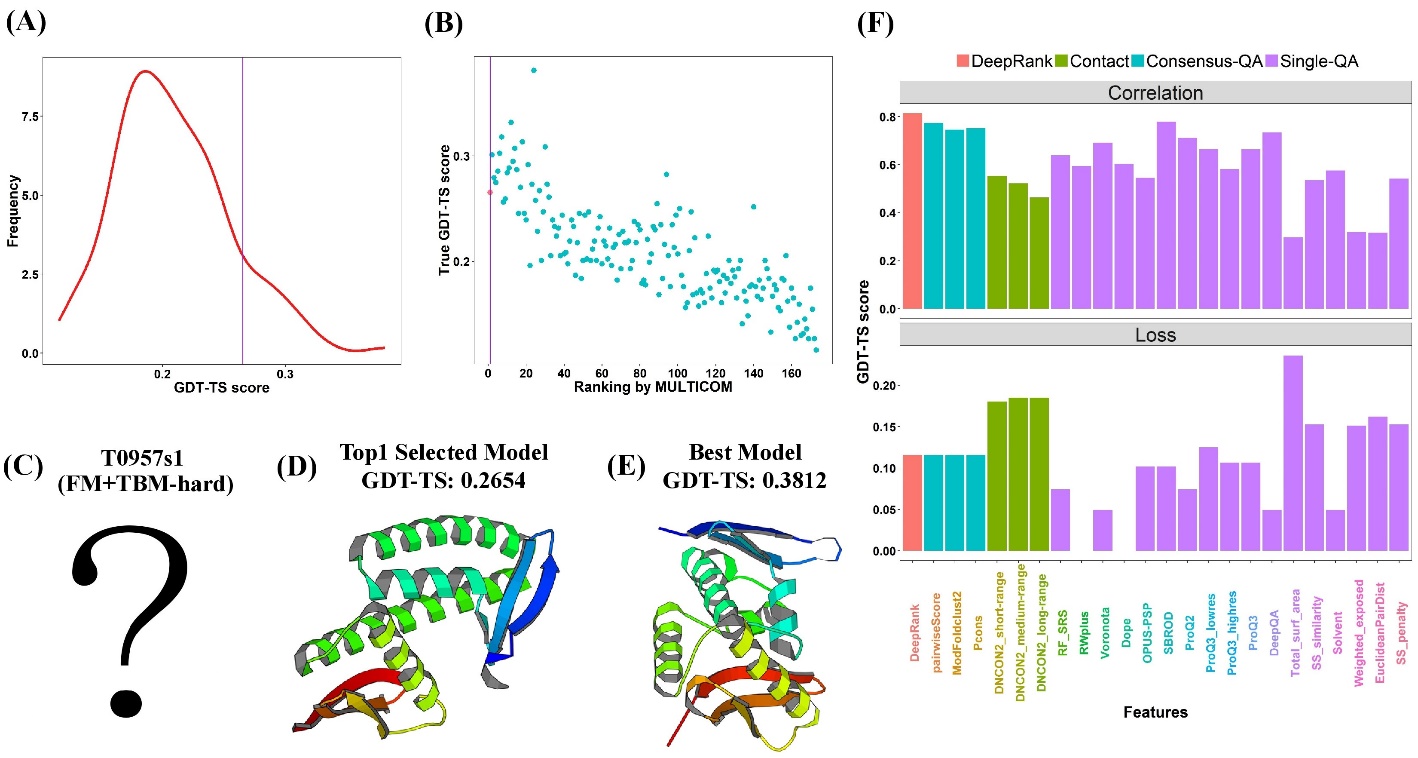


**Figure S17.** DeepRank model quality assessment for T0957s1. **(A)** The distribution of GDT-TS scores of 173 server models. **(B)** The plot of the true GDT-TS scores of models against their predicted ranking by MULTICOM. The point highlighted in red is the top model selected by DeepRank. **(C)** The experimental structure of target T0957s1 was not officially released to date. **(D)** The top selected model. **(E)** The best server model. **(F)** The ranking of individual QA methods for target T0957s1.


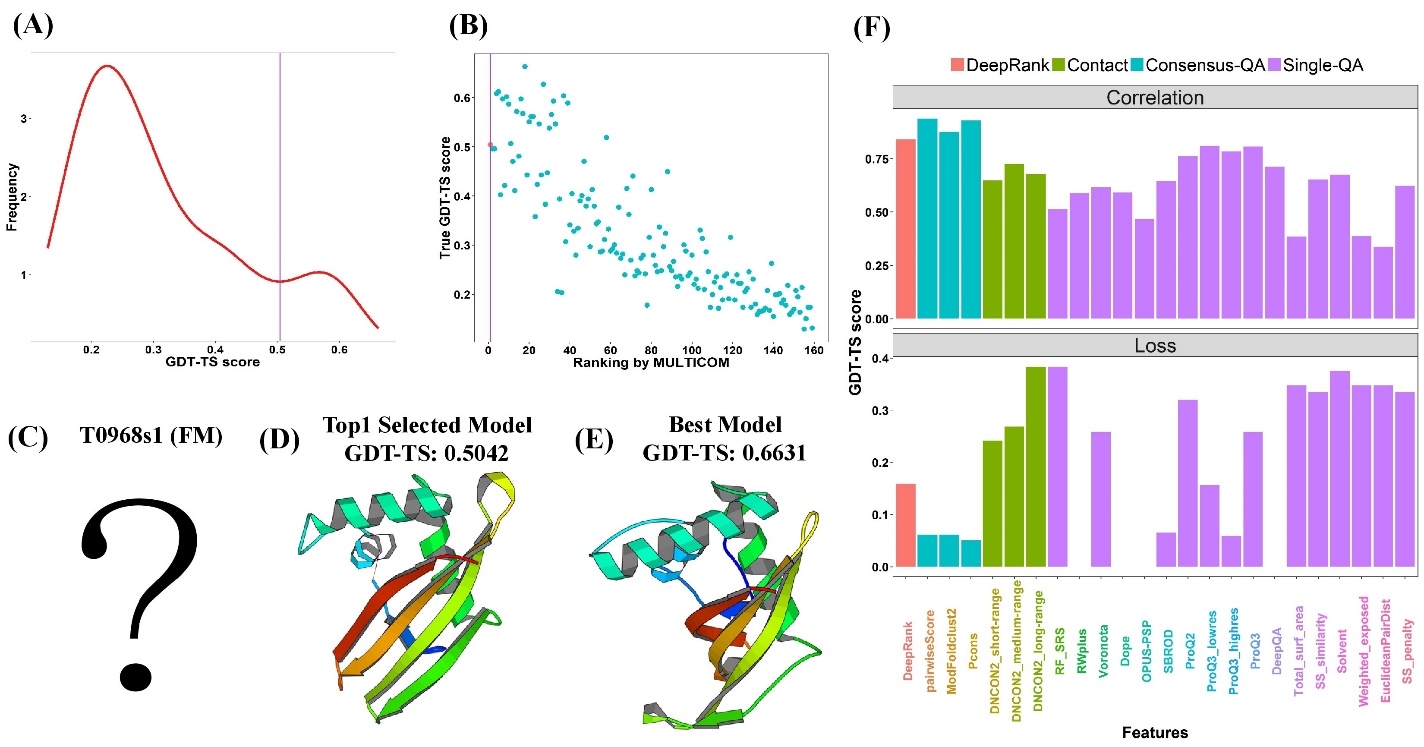


**Figure S18.** DeepRank model quality assessment for T0968s1. **(A)** The distribution of GDT-TS scores of 159 server models. **(B)** The plot of the true GDT-TS scores of models against their predicted ranking by MULTICOM. The point highlighted in red is the top model selected by DeepRank. **(C)** The experimental structure of target T0968s1 was not officially released to date. **(D)** The top selected model. **(E)** The best server model. **(F)** The ranking of individual QA methods for target T0968s1.


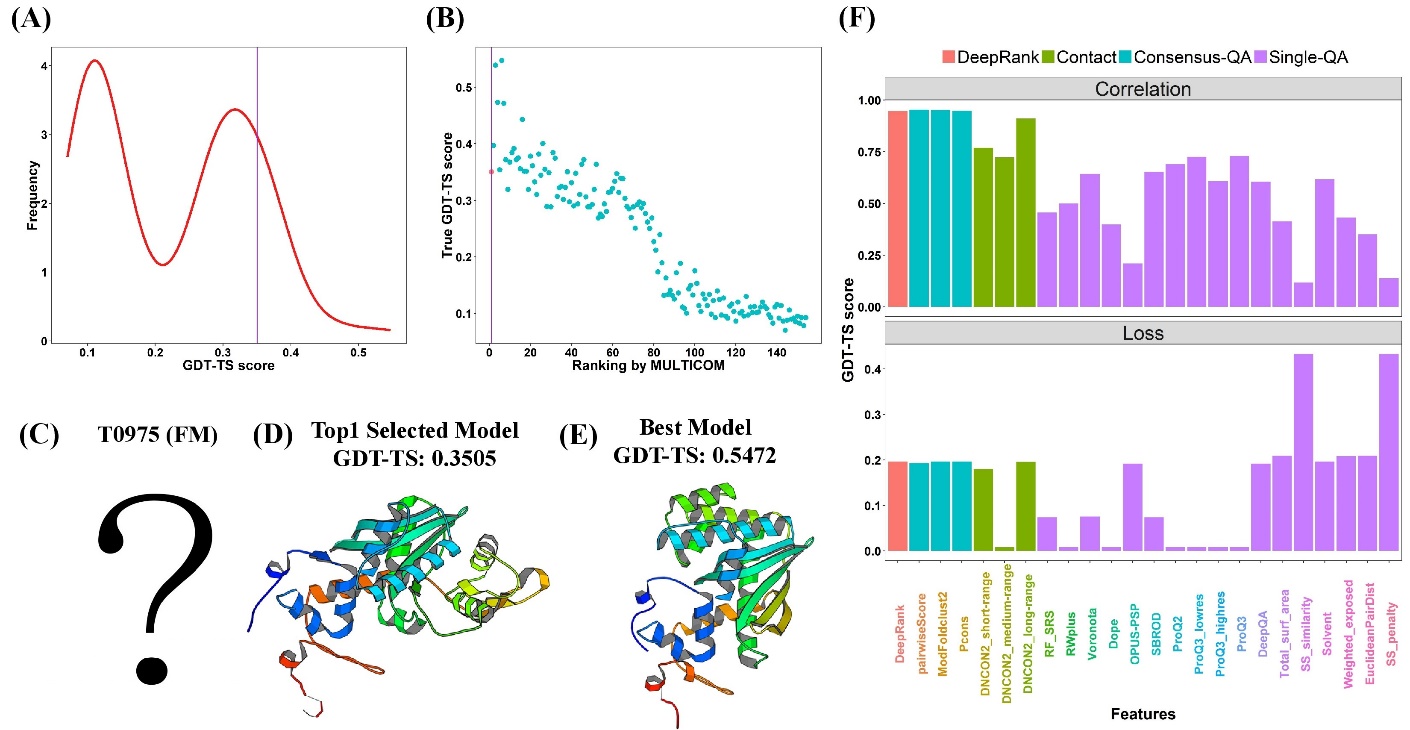


**Figure S19.** DeepRank model quality assessment for T0975. **(A)** The distribution of GDT-TS scores of 154 server models. **(B)** The plot of the true GDT-TS scores of models against their predicted ranking by MULTICOM. The point highlighted in red is the top model selected by DeepRank. **(C)** The experimental structure of target T0975 was not officially released to date. **(D)** The top selected model. **(E)** The best server model. **(F)** The ranking of individual QA methods for target T0975.


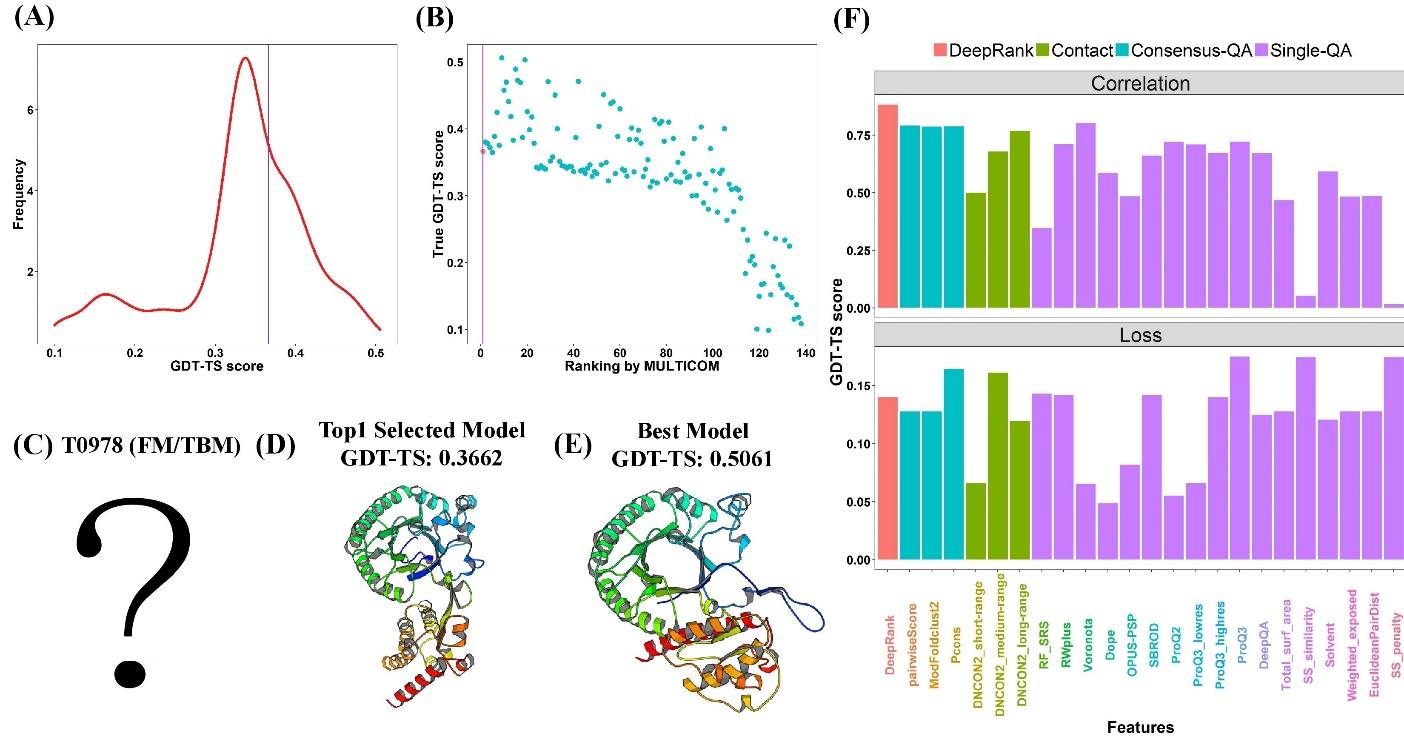


**Figure S20.** DeepRank model quality assessment for T0978. **(A)** The distribution of GDT-TS scores of 138 server models. **(B)** The plot of the true GDT-TS scores of models against their predicted ranking by MULTICOM. The point highlighted in red is the top model selected by DeepRank. **(C)** The experimental structure of target T0978 was not officially released to date. **(D)** The top selected model. **(E)** The best server model. **(F)** The ranking of individual QA methods for target T0978.


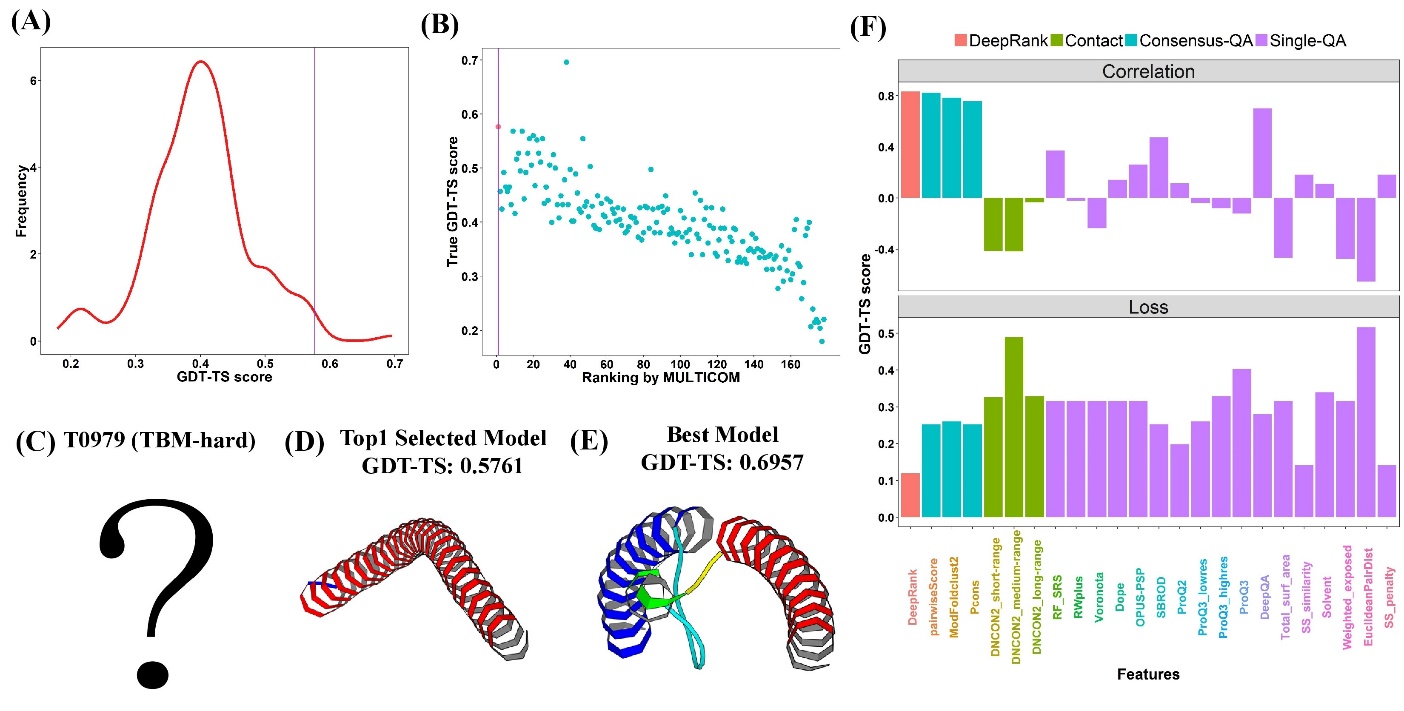


**Figure S21.** DeepRank model quality assessment for T0979. **(A)** The distribution of GDT-TS scores of 178 server models. **(B)** The plot of the true GDT-TS scores of models against their predicted ranking by MULTICOM. The point highlighted in red is the top model selected by DeepRank. **(C)** The experimental structure of target T0979 was not officially released to date. **(D)** The top selected model. **(E)** The best server model. **(F)** The ranking of individual QA methods for target T0979.


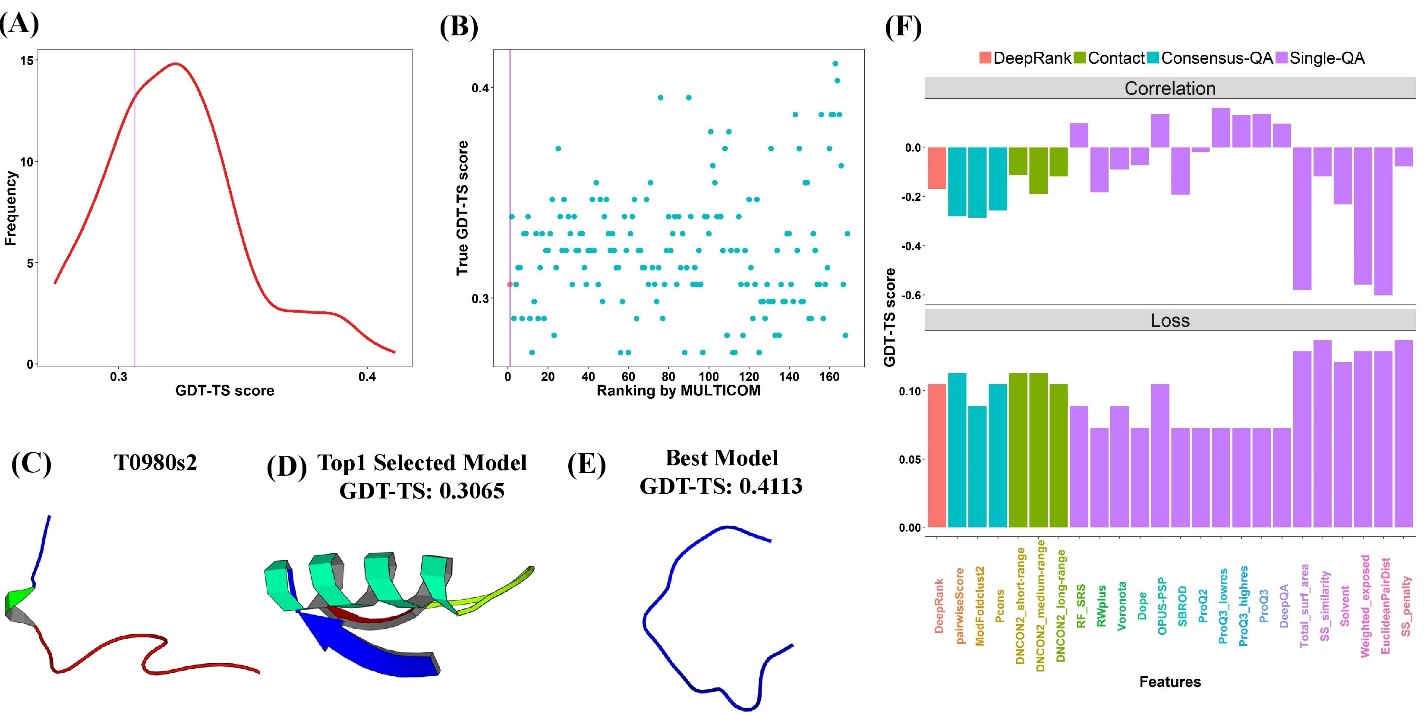


**Figure S22.** DeepRank model quality assessment for T0980s2. **(A)** The distribution of GDT-TS scores of 169 server models. **(B)** The plot of the true GDT-TS scores of models against their predicted ranking by MULTICOM. The point highlighted in red is the top model selected by DeepRank. **(C)** The native structure of target T0980s2 (PDB code: 6qnx). **(D)** The top selected model. **(E)** The best server model. **(F)** The ranking of individual QA methods for target T0980s2.


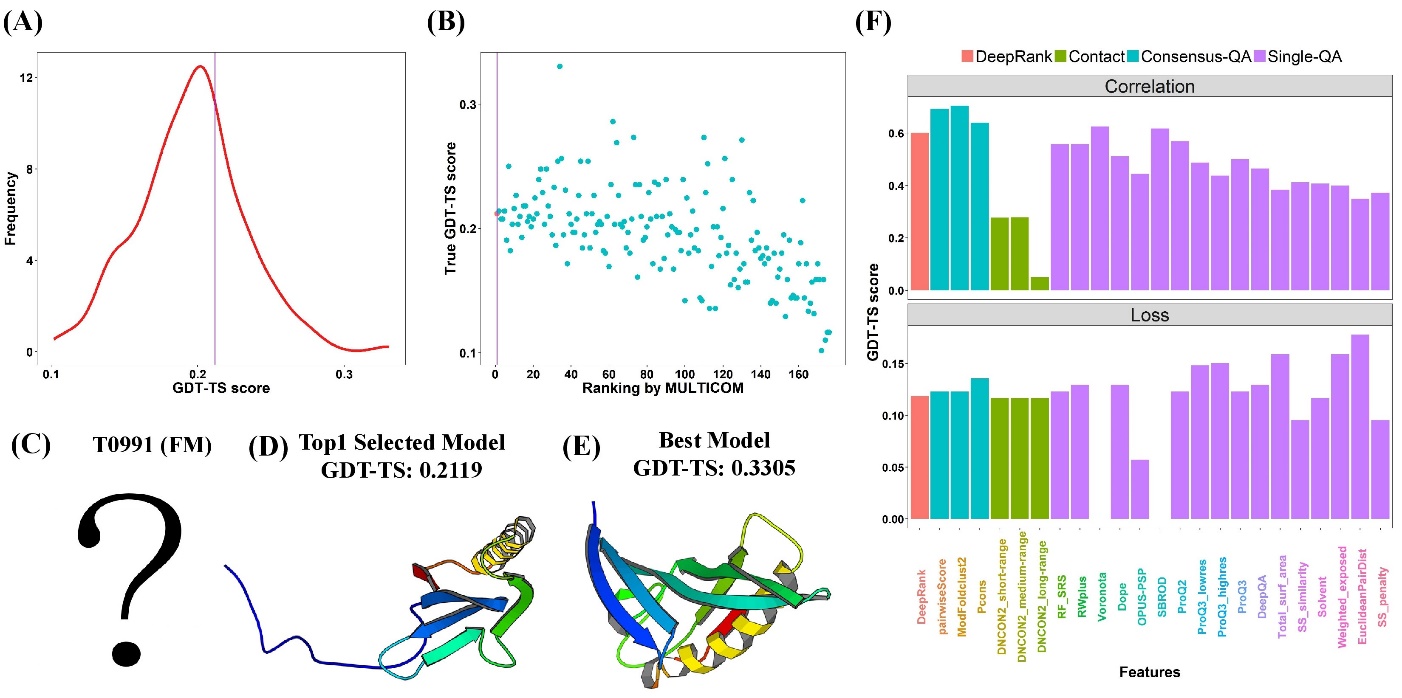


**Figure S23.** DeepRank model quality assessment for T0991. **(A)** The distribution of GDT-TS scores of 176 server models. **(B)** The plot of the true GDT-TS scores of models against their predicted ranking by MULTICOM. The point highlighted in red is the top model selected by DeepRank. **(C)** The experimental structure of target T0991 was not officially released to date. **(D)** The top selected model. **(E)** The best server model. **(F)** The ranking of individual QA methods for target T0991.


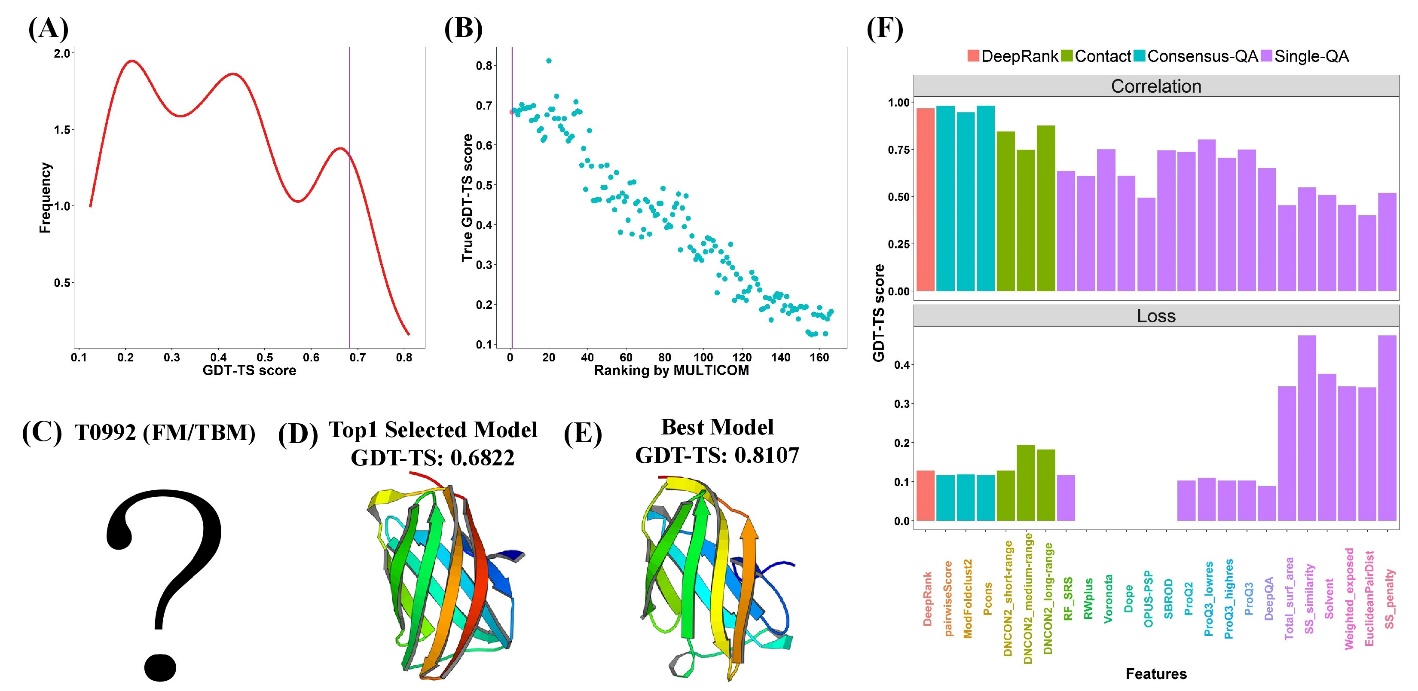


**Figure S24.** DeepRank model quality assessment for T0992. **(A)** The distribution of GDT-TS scores of 166 server models. **(B)** The plot of the true GDT-TS scores of models against their predicted ranking by MULTICOM. The point highlighted in red is the top model selected by DeepRank. **(C)** The experimental structure of target T0992 was not officially released to date. **(D)** The top selected model. **(E)** The best server model. **(F)** The ranking of individual QA methods for target T0992.


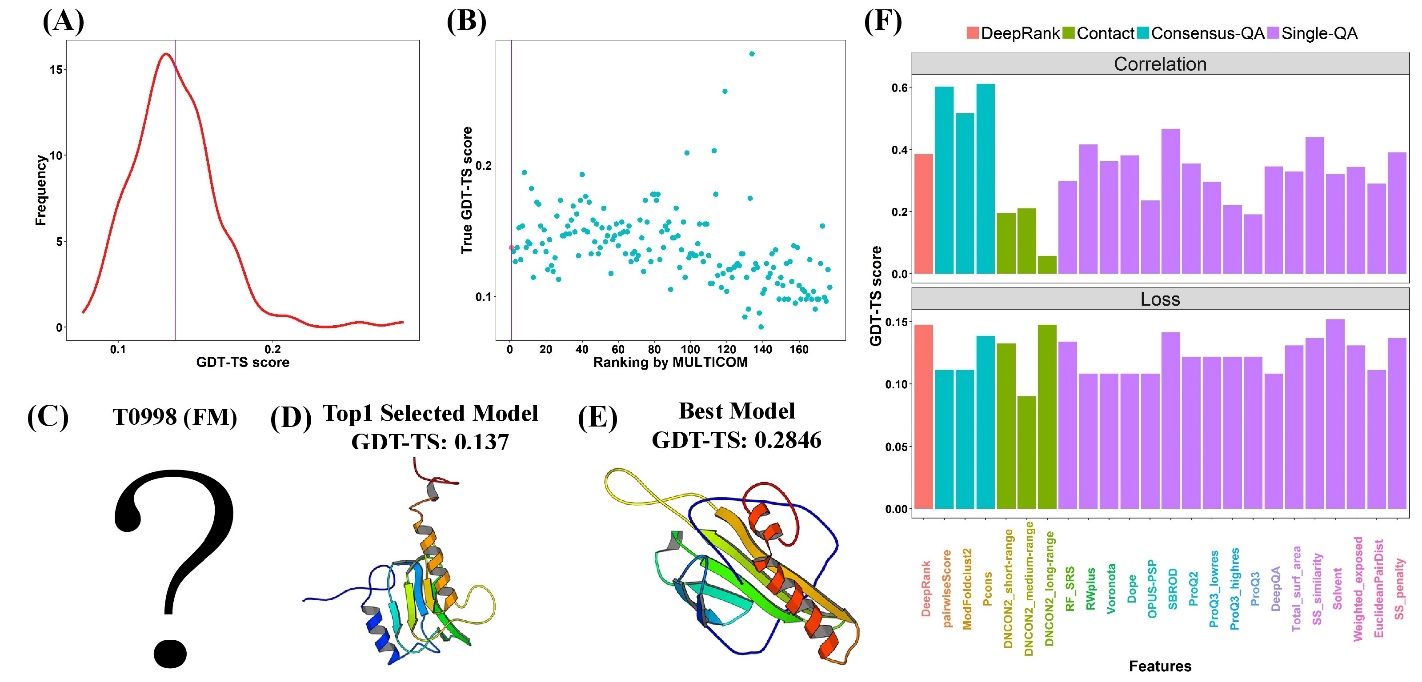


**Figure S25.** DeepRank model quality assessment for T0998. **(A)** The distribution of GDT-TS scores of 177 server models. **(B)** The plot of the true GDT-TS scores of models against their predicted ranking by MULTICOM. The point highlighted in red is the top model selected by DeepRank. **(C)** The experimental structure of target T0998 was not officially released to date. **(D)** The top selected model. **(E)** The best server model. **(F)** The ranking of individual QA methods for target T0998.


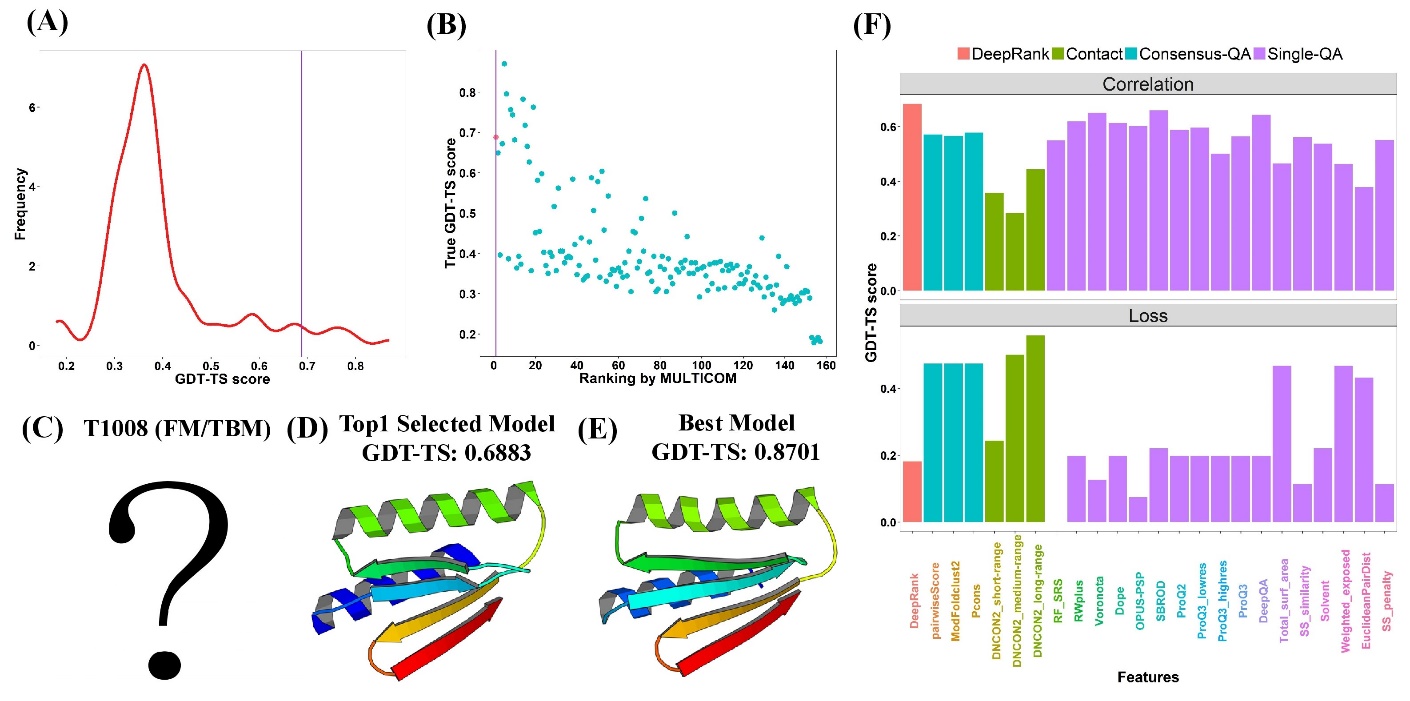


**Figure S26.** DeepRank model quality assessment for T1008. **(A)** The distribution of GDT-TS scores of 157 server models. **(B)** The plot of the true GDT-TS scores of models against their predicted ranking by MULTICOM. The point highlighted in red is the top model selected by DeepRank. **(C)** The experimental structure of target T1008 was not officially released to date. **(D)** The top selected model. **(E)** The best server model. **(F)** The ranking of individual QA methods for target T1008.


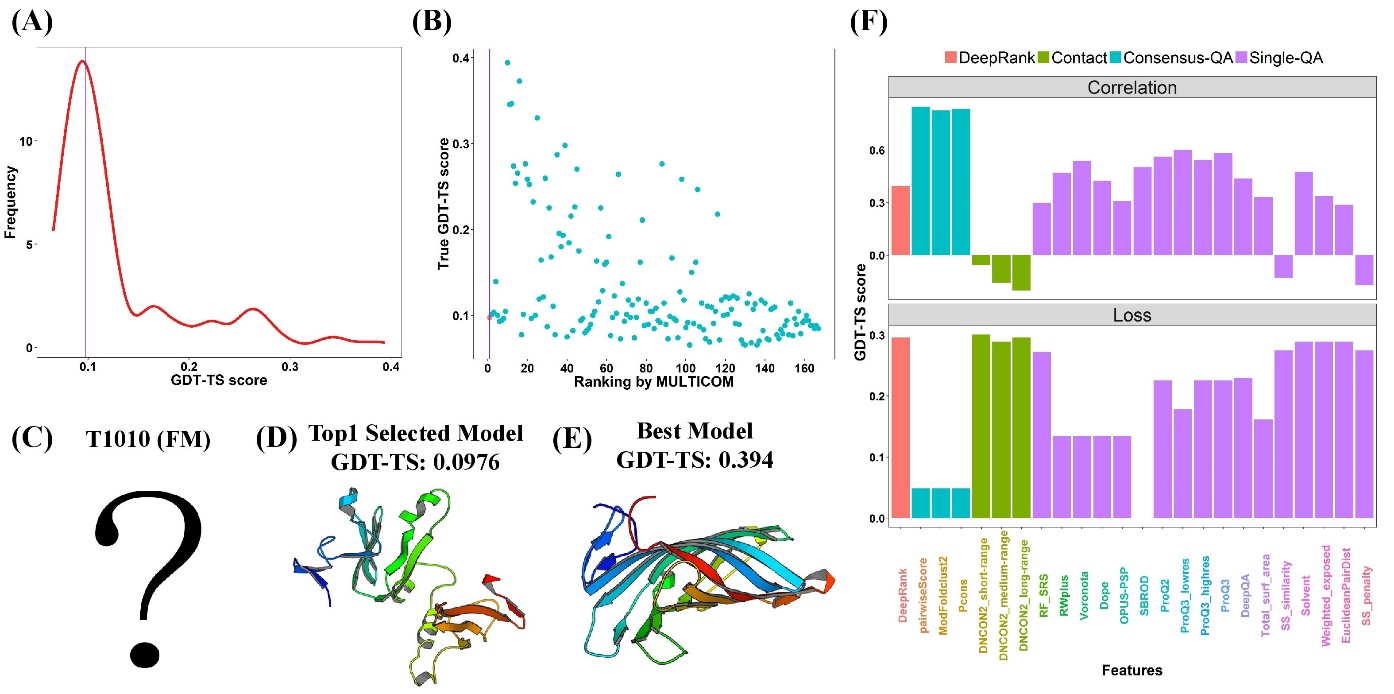


**Figure S27.** DeepRank model quality assessment for T1010. **(A)** The distribution of GDT-TS scores of 167 server models. **(B)** The plot of the true GDT-TS scores of models against their predicted ranking by MULTICOM. The point highlighted in red is the top model selected by DeepRank. **(C)** The experimental structure of target T1010 was not officially released to date. **(D)** The top selected model. **(E)** The best server model. **(F)** The ranking of individual QA methods for target T1010.


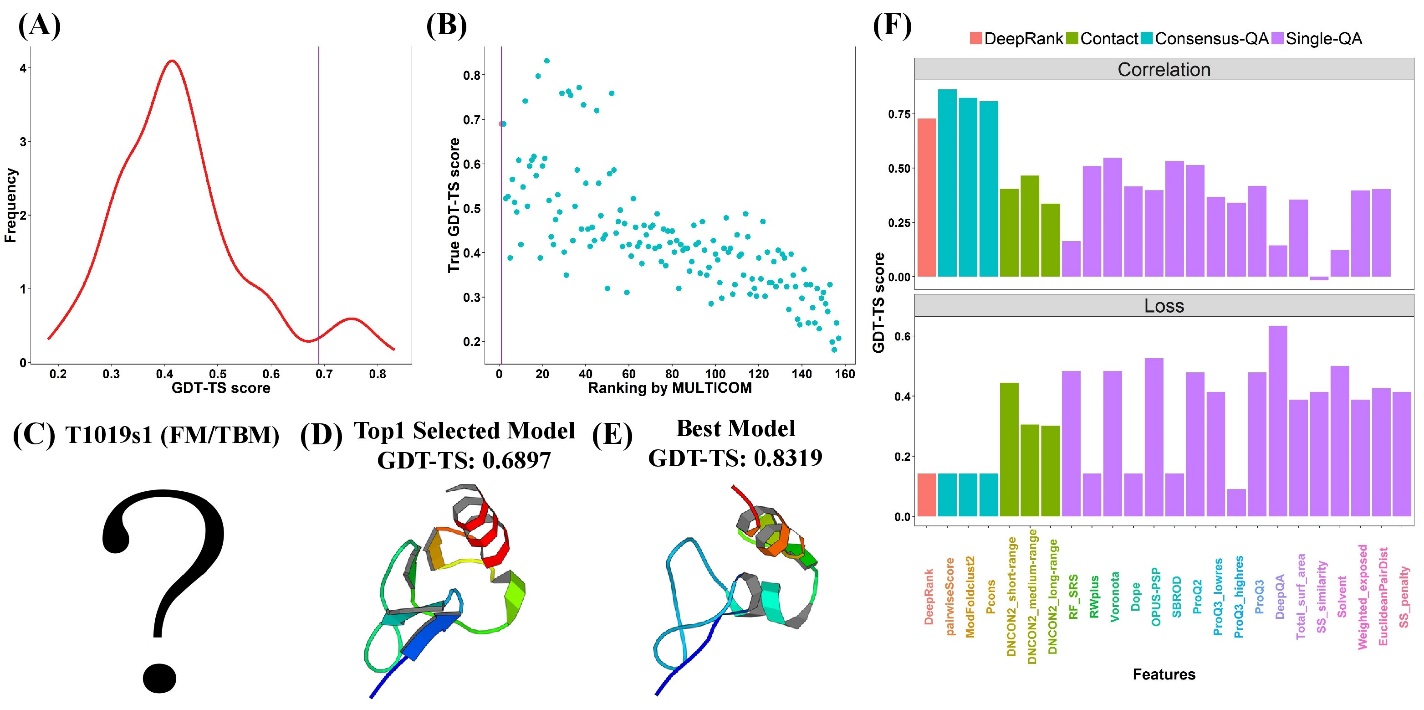


**Figure S28.** DeepRank model quality assessment for T1019s1. **(A)** The distribution of GDT-TS scores of 157 server models. **(B)** The plot of the true GDT-TS scores of models against their predicted ranking by MULTICOM. The point highlighted in red is the top model selected by DeepRank. **(C)** The experimental structure of target T1019s1 was not officially released to date. **(D)** The top selected model. **(E)** The best server model. **(F)** The ranking of individual QA methods for target T1019s1.


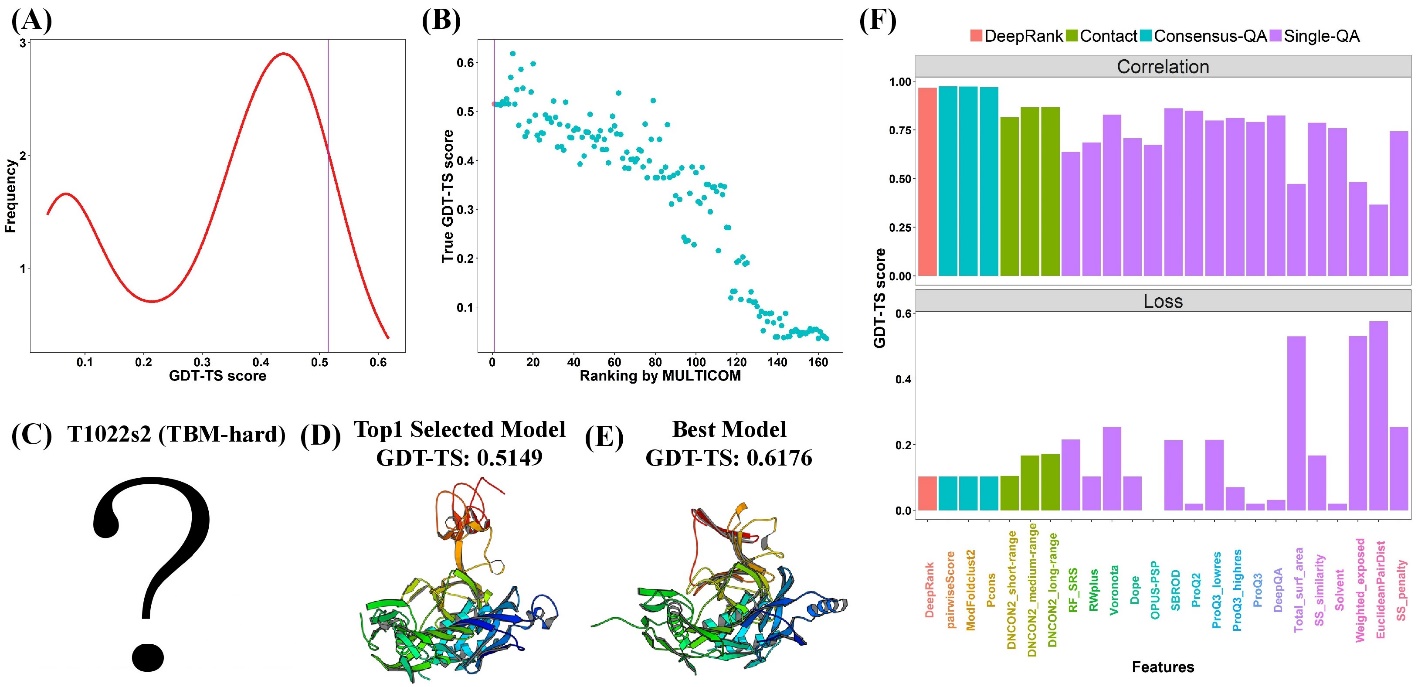


**Figure S29.** DeepRank model quality assessment for T1022s2. **(A)** The distribution of GDT-TS scores of 164 server models. **(B)** The plot of the true GDT-TS scores of models against their predicted ranking by MULTICOM. The point highlighted in red is the top model selected by DeepRank. **(C)** The experimental structure of target T1022s2 was not officially released to date. **(D)** The top selected model. **(E)** The best server model. **(F)** The ranking of individual QA methods for target T1022s2.

**Reference**

1. Cheng, J.J.B.s.b., *A multi-template combination algorithm for protein comparative modeling.* 2008. **8**(1): p. 18.

2. Wang, Z., J. Eickholt, and J.J.B. Cheng, *APOLLO: a quality assessment service for single and multiple protein models.* 2011. **27**(12): p. 1715-1716.
